# A Leakage-Aware Benchmark Study of Machine Learning Models for Deep Eutectic Solvent Property Prediction

**DOI:** 10.1021/acsomega.6c05807

**Published:** 2026-08-07

**Authors:** Hakim Faraji, Julio Brito Santana, Ruth Rodríguez-Ramos, Bárbara Socas-Rodríguez, Antonio V. Herrera Herrera

**Affiliations:** † Departamento de Química, Área de Química Analítica, Facultad de Ciencias, Universidad de La Laguna (ULL), Tenerife 38200, Spain; ‡ Departamento de Ingeniería Informática y de Sistemas, Universidad de La Laguna (ULL), Tenerife 38200, Spain; § CIMO, LA SusTEC, Instituto Politécnico de Bragança, Campus de Santa Apolónia, Bragança 5300-253, Portugal

## Abstract

Predicting the physicochemical properties of deep eutectic solvents (DESs) remains challenging due to the large combinatorial design space and the complex, composition- and temperature-dependent interactions governing their behavior. While machine learning (ML) has been widely applied to DES property prediction, reported performance is often sensitive to data set structure, feature representation, and validation design, raising questions about the reliability and transferability of existing models. In this work, we present a systematic evaluation of descriptor-based ML models for DES property prediction using a curated and high-confidence data set spanning 2003–2026. A unified feature representation combining molecular descriptors, molar composition, and temperature is employed, and model performance is assessed under a hierarchy of validation protocols designed to control for data leakage and progressively increase extrapolation difficulty. The results show that predictive performance is strongly dependent on the validation design. Density and refractive index exhibit relatively stable behavior, while surface tension shows moderate predictability. In contrast, electrical conductivity and viscosity display a strong dependence on temperature and limited contribution from descriptor-based features. Under extrapolative validation, performance for these properties deteriorates substantially, indicating limited transferability. Additional analyses based on feature-space distance and similarity-based baselines indicate that prediction errors are strongly associated with training-domain proximity and that local similarity in the current feature space is insufficient for reliable prediction outside observed data regions. These findings suggest that the primary limitation arises from the representational capacity of static descriptor-based features rather than model choice alone. Overall, this study provides a structured and reproducible assessment of the conditions under which descriptor-based ML models can be expected to succeed or fail in DES systems and highlights the importance of rigorous, leakage-aware evaluation in data-driven chemical modeling.

## Graphical abstract

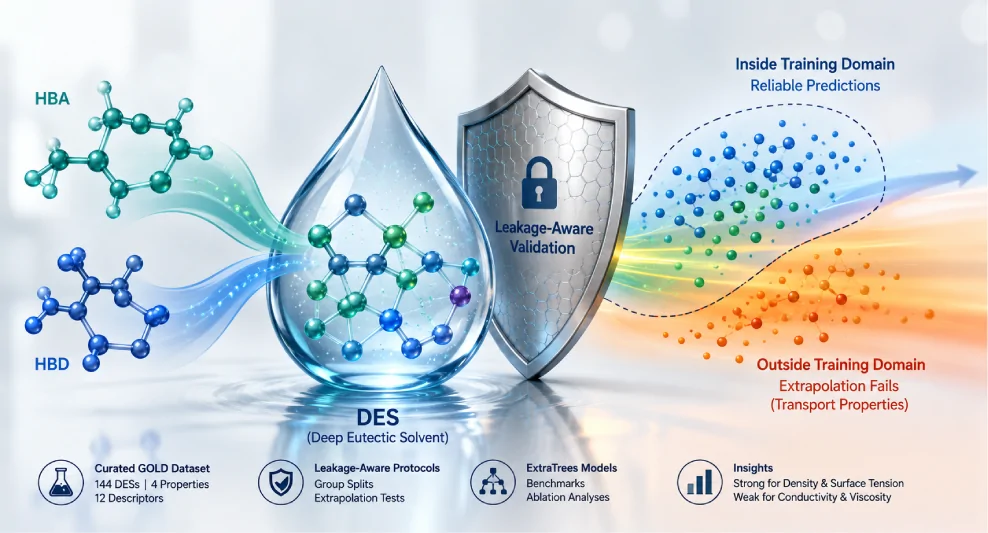

## Introduction

Deep eutectic solvents (DESs) have attracted considerable attention as low-cost and environmentally benign alternatives to conventional solvents and ionic liquids since their introduction by Abbott and coworkers.^1^ Formed through the combination of a hydrogen bond acceptor (HBA) and a hydrogen bond donor (HBD), DES systems exhibit highly tunable physicochemical properties, including density, viscosity, electrical conductivity, and polarity, making them attractive for applications in green chemistry, extraction, catalysis, and materials processing.^2^

Despite extensive experimental and theoretical efforts, the rational design of DES with targeted properties remains a major challenge. This difficulty arises from the vast combinatorial space of possible HBA–HBD combinations and the strong dependence of macroscopic properties on composition, temperature, and complex intermolecular interactions. In particular, transport properties such as viscosity and electrical conductivity are governed by collective phenomena, including hydrogen-bonding networks and microstructural organization, which are inherently nonlinear and difficult to describe using conventional thermodynamic or semiempirical models.^3,4^

In recent years, machine learning (ML) has emerged as a promising approach for DES property prediction by leveraging data-driven relationships between composition and physicochemical behavior.^5^ A range of descriptor-based representations and regression models have been explored, including ensemble methods and gradient boosting algorithms applied to properties such as viscosity and conductivity.^6,7^ These studies demonstrate the potential of ML for capturing structure–property relationships in DES systems, particularly under interpolation-like conditions. However, it remains unclear whether reported model performance reflects genuinely transferable structure–property learning or is influenced by data set-specific biases and evaluation design.

Beyond individual property-specific models, several recent studies have highlighted the growing maturity of data-driven DES-property prediction. Halder et al. developed QSPR models for DES surface tension and explicitly examined mixture- and compound-level validation strategies, illustrating the importance of chemically meaningful data splitting in mixture-property modeling.^8^ Odegova et al. introduced DESignSolvents, an open platform for searching and predicting DES physicochemical properties, including melting temperature, density, and viscosity, using supervised machine learning models.^9^ Other studies have explored artificial neural networks and COSMO-RS-derived descriptors for DES surface tension, conductivity, and related thermophysical properties.^10,11^ These contributions demonstrate the increasing availability of DES-oriented ML tools while also emphasizing that model reliability depends strongly on feature representation, data set structure, and validation design.

Despite these advances, several critical challenges remain insufficiently addressed in the current literature. First, available data sets are often heterogeneous and chemically imbalanced, with a limited number of well-studied components dominating the majority of reported measurements. Second, commonly used feature representations rely on static molecular descriptors combined with composition and temperature variables, which provide only an approximate description of the dynamic and many-body interactions governing DES behavior. Third, and most importantly, model evaluation is frequently performed using validation schemes that do not adequately reflect extrapolative scenarios, making it difficult to assess the true generalization capabilities of descriptor-based models.

A further challenge arises from the diversity of DES formulations reported in the literature. Binary HBA–HBD systems, ternary DESs, hydrated DESs, and water-containing mixtures are often discussed under the broader DES terminology, but they introduce different compositional degrees of freedom and may not be interchangeable for ML validation. Ternary DESs require explicit treatment of additional components and composition variables, whereas water-containing DES systems can display substantially altered physicochemical behavior, particularly for viscosity and density.^12,13^ In the present study, the frozen GOLD data set is restricted to records that can be consistently represented by a defined HBA, HBD, molar ratio, temperature, and target property. This controlled scope enables leakage-aware benchmarking, but it also means that extrapolation to ternary or explicitly water-containing DES formulations should be regarded as outside the validated domain of the present descriptor representation.

The validation problem is closely related to the distinction between points-out and compounds-out evaluation in QSPR modeling of mixtures. Points-out validation evaluates interpolation among individual measurements, whereas compounds-out validation tests whether a model can generalize to chemically unseen components. For DES systems, this distinction is particularly important because repeated measurements of similar HBA–HBD combinations across temperature and composition can produce optimistic performance estimates if not explicitly controlled. Related mixture-QSPR studies have emphasized the importance of chemically meaningful validation when evaluating nonadditive mixture properties.^14^

These limitations raise a fundamental question: to what extent do descriptor-based ML models provide genuinely transferable predictive capabilities for DES properties beyond the domain of the training data?

In this work, we address this question by systematically examining the behavior of descriptor-based ML models for DES property prediction under controlled and leakage-aware conditions. Rather than focusing solely on predictive performance, the study aims to evaluate the reliability, limitations, and transferability of such models across different physicochemical properties.

By combining curated experimental data, a unified feature representation, and a hierarchy of validation strategies, this work provides a structured assessment of when descriptor-based approaches can be expected to succeed and where they remain limited. The results contribute to a clearer understanding of the capabilities and boundaries of data-driven modeling in complex chemical systems, such as DES.

## Materials and Methods

### Data Set Construction and Freezing

A unified data set of DES physicochemical properties was assembled from published experimental studies spanning the period 2003–2026. The data set integrates a review-based collection of earlier studies and a systematically screened set of recent literature, ensuring consistent coverage of component identity, molar composition, temperature, and property values.

To enable controlled and reproducible analysis, a high-confidence subset (GOLD) was defined, including only records with complete and internally consistent information. All modeling and evaluation in this study are based exclusively on this data set, which was treated as frozen after construction to prevent preprocessing drift and inadvertent data leakage. A summary of data set coverage across properties is provided in Table 1.

**Table 1:** Data Set Overview for the Frozen GOLD Modeling Data Set^a^

**Property**	***N***	**Temperature range (K)**	**Ratio range**(HBD/HBA)	**Unique HBA**	**Unique HBD**	**Unique**HBA–HBD pairs
Density	1024	273.15–363.15	0.09–20.00	79	97	223
Viscosity	944	273.15–368.15	0.09–20.00	94	104	257
Conductivity	545	278.15–343.15	0.09–20.00	44	55	128
Surface tension	469	288.15–353.15	0.09–20.00	36	47	90
Refractive index	511	288.15–353.00	0.09–20.00	39	57	119

a Property-specific sample size and coverage of temperature, composition, component diversity, and HBA–HBD pair diversity were used in the final audited workflow. Temperature ranges were calculated from reported measurement temperatures after conversion to Kelvin; composition ranges are reported as parsed HBD/HBA molar ratios.

Full details of data extraction, standardization, component normalization, and quality control procedures are provided in the Supporting Information (Sections S1–S2).

### Feature Representation

A unified descriptor-based representation was constructed to encode the DES composition and thermodynamic conditions. Molecular descriptors for individual HBA and HBD components were computed from canonical SMILES representations using RDKit. The descriptor set included physicochemical and topological descriptors, such as molecular weight, atom counts, heteroatom counts, hydrogen bond donor and acceptor counts, topological polar surface area, logP, molar refractivity, rotatable-bond count, ring counts, fraction of sp^3^ carbons, formal charge, and selected fragment-based indicators.

For each DES record, HBA and HBD descriptors were combined with composition-aware variables derived from the reported HBA/HBD molar ratio and temperature as an explicit numerical variable. This formulation provides a static, chemically derived descriptor representation of DES systems. It captures component identity, molecular structure, composition, and temperature while remaining an approximate representation of the dynamic and collective intermolecular interactions that govern DES behavior.

Feature construction was performed consistently across all property-specific data sets. Constant and near-constant descriptors were removed, missing numerical values were imputed within each training fold, and feature scaling was applied where required by the model class. To ensure leakage-safe modeling, target variables, nontarget property columns, source identifiers, and free-text metadata were excluded from the feature matrix before model training. Detailed descriptor definitions, feature construction rules, preprocessing steps, and the leakage-safe feature audit are provided in Sections S3–S5 of the Supporting Information. Additional analysis of data set coverage, component imbalance, and thermodynamic distribution is provided in Section S4.

### Modeling Approach

Property-specific regression models were developed for the density, viscosity, electrical conductivity, surface tension, and refractive index. Three model classes were selected to represent increasing model complexity while maintaining interpretability and computational reproducibility: ridge regression as a linear regularized baseline, extra trees regression as the primary nonlinear ensemble model, and histgradient boosting regression as an additional gradient-boosting benchmark.

All models were implemented as reproducible Scikit-learn pipelines. Numerical features were median-imputed within each training fold to prevent information transfer from the test set. Standardization was applied to linear models and distance-sensitive analyses, whereas tree-based models were trained on an imputed descriptor matrix. ExtraTrees was used as the primary reported model because it provided stable performance across property-specific data sets, handled nonlinear descriptor interactions, and remained computationally efficient under repeated group-aware validation.

Hyperparameters were specified using fixed, default-informed configurations rather than being optimized separately on the test folds. This choice was made to reduce the risk of overfitting validation protocols and to ensure that differences in performance primarily reflected data set structure, feature representation, and validation design rather than extensive model tuning. The full stage-specific model configurations used for diagnostic analysis, leakage-safe validation, feature ablation, applicability domain analysis, and interpretability are summarized in Table S8.

### Validation Protocols and Leakage Control

Model performance was evaluated under a hierarchy of validation protocols designed to distinguish interpolation-driven performance from extrapolative generalization. Four protocols were defined as follows:

• Protocol A (old-like/diagnostic): reproduces the behavior of the original pipeline for forensic comparison and potential leakage identification. This protocol is closest to a points-out or measurement-level split and is used only diagnostically; it is not considered a valid estimate of transferable model performance.

• Protocol B (leakage-corrected pair + ratio grouping): removes all target and cross-property information from the feature matrix and groups samples by HBA–HBD ratios. This prevents repeated measurements of the same DES composition from appearing in both training and test sets and represents a composition-aware, leakage-safe split.

• Protocol C (strict HBA–HBD pair grouping): groups samples at the HBA–HBD pair level, excluding pair overlap between training and test sets. This protocol corresponds to a pair-out or mixture-out validation setting and evaluates whether models can generalize across compositions without reusing the same component pair.

• Protocol D (leave-component-out): excludes entire HBA or HBD components from the training set and evaluates predictions only on systems containing unseen components. This protocol is closest to a compounds-out validation strategy and provides the most stringent assessment of chemical extrapolation.^14^

These protocols progressively increase the level of generalization difficulty, enabling differentiation between interpolation within observed chemical neighborhoods and extrapolation to unseen chemical spaces. A summary of the validation design is provided in Table 2.

**Table 2:**
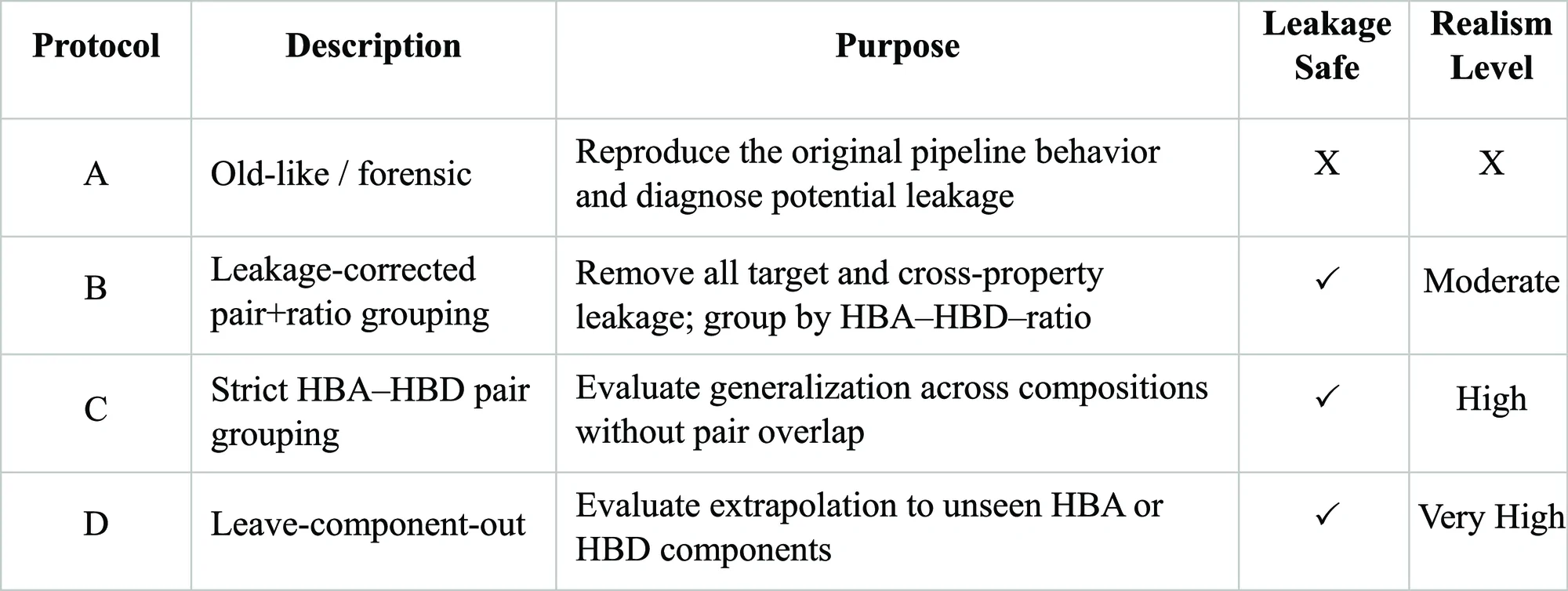
Validation Protocols Used for Leakage and Generalization Audit

A formal leakage audit was applied to all of the leakage-safe workflows. The audit verified that the target property was not present in the feature matrix, that nontarget property columns were excluded, and that grouping variables were used only for data splitting rather than as predictors unless explicitly evaluated as part of a baseline model. Implementation details, grouping definitions, and leakage audit outputs are provided in Section S5.

### Baseline Models

To assess whether descriptor-based ML models provide predictive value beyond simple thermodynamic and compositional trends, several baseline models were evaluated under the same validation protocols as those used for the full models. These included a mean predictor, a temperature-only model, a ratio-only model, a temperature + ratio model, and a component-identity baseline.

The temperature-only and ratio-based baselines quantify the extent to which the predictive performance can be explained by simple measurement conditions or composition variables. The component-identity baseline provides a conservative estimate of performance driven by repeated chemical identities rather than transferable-descriptor information. Comparing the full descriptor-based model against these baselines enables the direct estimation of the incremental value of molecular descriptors under leakage-safe validation. Full baseline implementation details are provided in Section S6.

### Feature Ablation Analysis

To evaluate the contribution of different feature groups, a structured ablation analysis was performed using progressively enriched feature sets: descriptors only, descriptors plus molar ratio information, descriptors plus temperature, descriptor interaction features, and the full descriptor–ratio–temperature representation.

This analysis was designed to determine whether predictive performance is driven primarily by molecular descriptors, composition, temperature, or their combined representation. It is particularly important for distinguishing genuine descriptor-driven structure–property learning from performance dominated by temperature trends. Detailed results and implementation are provided in Section S6.

### Viscosity-Specific Diagnostics

Given the known challenges associated with viscosity prediction, additional diagnostic analyses were performed for this property. These included a comparison of raw and transformed targets and an evaluation of error behavior under different validation settings.

These analyses are intended to distinguish between distributional effects and limitations related to the feature representation and generalization. Detailed procedures and results are provided in Section S6.

### Interpretability Analysis

Model interpretability was assessed using permutation feature importance and SHapley Additive Explanations (SHAP). These analyses were performed on leakage-safe models and were used to identify features that contributed most strongly to the in-domain model predictions.

Feature importance results were interpreted as supportive diagnostic evidence rather than as proof of extrapolative transferability. This distinction is important because a feature may be influential within the observed training domain while still failing to support reliable predictions for chemically unseen components. Additional methodological details and extended results are provided in Section S7. All scripts, frozen data sets, leakage-audit workflows, and reproducible figure/table generation pipelines associated with this study are publicly available in the accompanying repository and archived release described in the Supporting Information (Section S8) and at 10.5281/zenodo.21209405.

## Results and Discussion

### Data Set Structure, Chemical-Space Coverage, and Imbalance

Before evaluating model performance, we first examined the structure and chemical-space coverage of the frozen GOLD data set. The property-specific modeling subsets contain 1024 density records, 944 viscosity records, 545 electrical conductivity records, 469 surface tension records, and 511 refractive index records (Table 1). Component diversity varies across properties, with 36–94 unique HBAs, 47–104 unique HBDs, and 90–257 unique HBA–HBD pairs represented in the property-specific subsets. This coverage provides a useful basis for benchmarking descriptor-based models, but it remains uneven across both component identity and property type.

A quantitative imbalance analysis reveals a pronounced long-tail distribution of DES components (Figure S1 and Table S4). Across all property-specific records, the data set contains 99 unique HBAs, 122 unique HBDs, and 302 unique HBA–HBD pairs; however, a small number of components dominate the available measurements. The top ten HBAs account for 62.5% of all property-specific records, while the top ten HBDs account for 57.5%. This imbalance is further reflected in high Gini coefficients for both HBA and HBD frequency distributions, indicating that the chemical space is dominated by frequently studied components rather than uniformly sampled DES combinations.

The compositional and thermodynamic coverage is similarly nonuniform. The parsed HBD/HBA molar ratio spans 0.09–20.00, but the median ratio is 2.00 across the property-specific records, indicating concentration around commonly reported DES compositions. Temperature coverage spans 273.15–368.15 K overall, with a median temperature of 313.15 K, reflecting substantial sampling around near-ambient to moderately elevated conditions. These patterns are important because repeated measurements of similar component pairs across temperature and composition can inflate apparent model performance if validation protocols do not explicitly control for chemical overlap.

The present benchmark therefore focuses on a controlled and consistently encoded representation of DES records defined by HBA identity, HBD identity, molar ratio, temperature, and target property. Ternary DESs, hydrated DESs, and explicitly water-containing systems introduce additional compositional degrees of freedom and are not treated as interchangeable with the binary HBA–HBD representation used in the frozen GOLD benchmark. Consequently, extrapolation to such systems should be regarded as outside the validated domain of the present descriptor representation.

This data set structure motivates the use of group-aware and leave-component-out validation protocols. In particular, random or weakly constrained splits may test interpolation within densely sampled regions, whereas pair-level and component-level splits provide a more realistic assessment of whether descriptor-based models can generalize beyond previously observed chemical neighborhoods.

### Evaluation Strategy and Leakage-Aware Validation Design

The overall workflow of the present study is summarized in Figure 1. In contrast to conventional ML studies that primarily report predictive performance, the approach adopted here focuses on systematically evaluating model behavior under controlled and progressively more stringent validation conditions.

**Figure 1:**
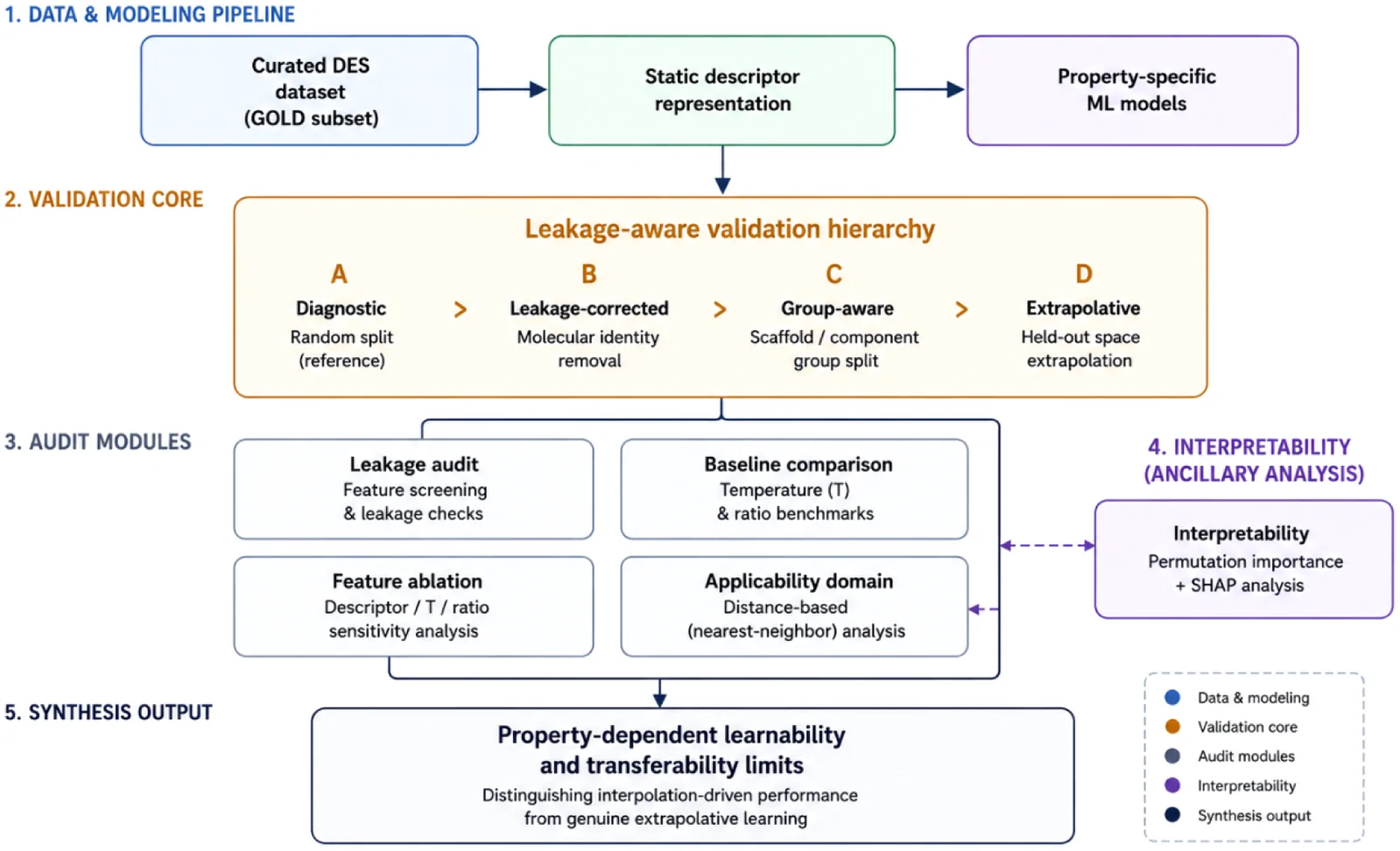
Overview of the leakage-aware DES machine learning audit framework.

Starting from a curated and frozen high-confidence data set (Table 1), models are evaluated under a hierarchy of validation protocols that explicitly control for data leakage and increasing levels of extrapolation (Table 2). In addition, complementary analyses—including baseline comparison, feature ablation, applicability-domain analysis, nearest-neighbor benchmarking, and interpretability—are integrated into the evaluation pipeline to distinguish between apparent predictive accuracy and genuinely transferable structure–property relationships.

The four validation protocols can be interpreted in relation to established validation paradigms in mixture-QSPR modeling. Protocol A is closest to a points-out or measurement-level split and is retained only for diagnostic purposes because it may allow chemically similar measurements to appear across training and test sets. Protocol B removes repeated HBA–HBD–ratio compositions across training and test sets and therefore represents a composition-aware leakage-safe split. Protocol C corresponds to a pair-out or mixture-out setting because HBA–HBD pairs are excluded from overlap between training and test sets. Protocol D is closest to a compounds-out validation strategy because entire HBA or HBD components are excluded from training and evaluated only at test time.

Such an evaluation strategy is particularly important in DES systems, where repeated measurements of similar compositions and shared component identities can introduce a systematic bias in performance estimation. Recent methodological studies have emphasized that an inadequate validation design can lead to overestimated predictive performance and misleading conclusions regarding model generalization.^5,15^ The analysis presented below therefore focuses not only on predictive performance but also on the reliability, robustness, and transferability of descriptor-based models across different DES properties.

### Validation Sensitivity and Extrapolative Generalization

The effect of the validation design on predictive performance is shown in Figure 2. A consistent trend is observed across all investigated properties: model performance decreases as validation protocols become progressively more stringent.

**Figure 2:**
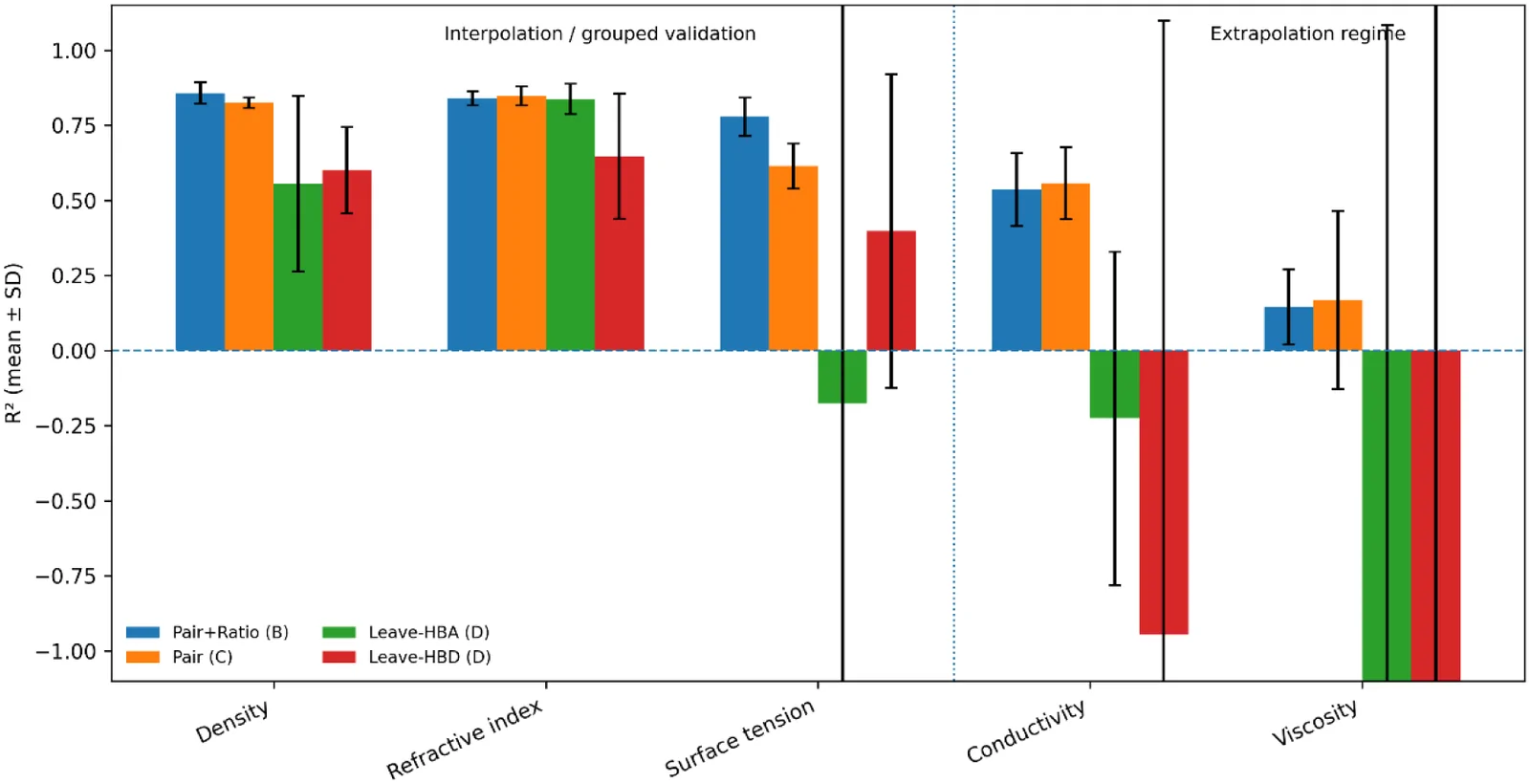
Validation sensitivity across DES property prediction tasks.

Under leakage-corrected pair + ratio validation (Protocol B), relatively high predictive performance is observed for density and refractive index, moderate performance for surface tension and electrical conductivity, and limited performance for viscosity. These results are in line with previous studies, suggesting that the density and refractive index can often be approximated as quasi-additive functions of component contributions.^16,17^

However, when moving from Protocol B to strict HBA–HBD pair grouping (Protocol C), performance decreases across most properties, indicating that models partially rely on compositional similarity between training and test data. This effect becomes more pronounced under leave-component-out validation (Protocol D), where entire HBA or HBD components are excluded from the training set. Quantitative performance metrics for leakage-safe validation protocols are summarized in Table 3.

**Table 3:** Model Performance of the Primary ExtraTrees Model under Leakage-Safe Validation Protocols^a^

**Property**	**Protocol**	**Model**	***R*** ^**2**^ **mean ± SD** (95% CI)	**MAE**mean ± SD (95% CI)
Density	Pair + Ratio (B)	ExtraTrees	0.857 ± 0.035 (95% CI: 0.826–0.888)	0.028 ± 0.004 (95% CI: 0.024–0.032)
Density	Pair (C)	ExtraTrees	0.825 ± 0.017 (95% CI: 0.810–0.840)	0.036 ± 0.002 (95% CI: 0.034–0.038)
Density	Leave-HBA (D)	ExtraTrees	0.555 ± 0.292 (95% CI: 0.374–0.736)	0.048 ± 0.008 (95% CI: 0.043–0.053)
Density	Leave-HBD (D)	ExtraTrees	0.600 ± 0.144 (95% CI: 0.511–0.689)	0.046 ± 0.011 (95% CI: 0.039–0.053)
Refractive index	Pair + Ratio (B)	ExtraTrees	0.839 ± 0.024 (95% CI: 0.818–0.860)	0.010 ± 0.002 (95% CI: 0.008–0.012)
Refractive index	Pair (C)	ExtraTrees	0.847 ± 0.032 (95% CI: 0.819–0.875)	0.010 ± 0.003 (95% CI: 0.007–0.013)
Refractive index	Leave-HBA (D)	ExtraTrees	0.837 ± 0.050 (95% CI: 0.806–0.868)	0.011 ± 0.002 (95% CI: 0.010–0.012)
Refractive index	Leave-HBD (D)	ExtraTrees	0.647 ± 0.208 (95% CI: 0.518–0.776)	0.011 ± 0.002 (95% CI: 0.010–0.012)
Surface tension	Pair + Ratio (B)	ExtraTrees	0.778 ± 0.064 (95% CI: 0.722–0.834)	3.339 ± 0.546 (95% CI: 2.860–3.818)
Surface tension	Pair (C)	ExtraTrees	0.614 ± 0.075 (95% CI: 0.548–0.680)	5.010 ± 0.529 (95% CI: 4.546–5.474)
Surface tension	Leave-HBA (D)	ExtraTrees	–0.176 ± 1.465 (95% CI: −1.084 to 0.732)	5.378 ± 1.725 (95% CI: 4.309–6.447)
Surface tension	Leave-HBD (D)	ExtraTrees	0.397 ± 0.522 (95% CI: 0.073–0.721)	5.147 ± 1.012 (95% CI: 4.520–5.774)
Conductivity	Pair + Ratio (B)	ExtraTrees	0.535 ± 0.121 (95% CI: 0.429–0.641)	196.088 ± 83.394 (95% CI: 122.990–269.186)
Conductivity	Pair (C)	ExtraTrees	0.557 ± 0.120 (95% CI: 0.452–0.662)	190.280 ± 50.091 (95% CI: 146.373–234.187)
Conductivity	Leave-HBA (D)	ExtraTrees	–0.226 ± 0.555 (95% CI: −0.570 to 0.118)	241.915 ± 273.383 (95% CI: 72.470–411.360)
Conductivity	Leave-HBD (D)	ExtraTrees	–0.947 ± 2.044 (95% CI: –2.214–0.320)	278.615 ± 50.241 (95% CI: 247.475–309.755)
Viscosity	Pair + Ratio (B)	ExtraTrees	0.145 ± 0.125 (95% CI: 0.035–0.255)	1273.555 ± 170.710 (95% CI: 1123.921–1423.189)
Viscosity	Pair (C)	ExtraTrees	0.168 ± 0.296 (95% CI: –0.091–0.427)	1350.434 ± 948.323 (95% CI: 519.192–2181.676)
Viscosity	Leave-HBA (D)	ExtraTrees	–3.625 ± 4.708 (95% CI: −6.543 to −0.707)	2447.461 ± 1488.839 (95% CI: 1524.669–3370.253)
Viscosity	Leave-HBD (D)	ExtraTrees	–2.653 ± 4.624 (95% CI: −5.519 to 0.213)	3548.825 ± 1833.759 (95% CI: 2412.249–4685.401)

a Values are reported as mean ± SD with 95% confidence intervals (CI) calculated across repeated validation folds/runs. Extended relative-error metrics for transport properties are provided in Table S11.

The degradation in performance under Protocol D is strongly property-dependent. Density and refractive index retain moderate predictive capability, suggesting the presence of relatively stable structure–property relationships that can partially transfer across chemical space. Surface tension exhibits intermediate behavior, with partial retention of predictive performance.

In contrast, electrical conductivity and viscosity show a substantial decline in predictive accuracy under extrapolative conditions, with R^2^ values approaching zero or becoming negative in leave-component-out scenarios. This behavior indicates that a significant portion of the apparent model performance under less stringent protocols arises from interpolation within previously observed chemical neighborhoods rather than true extrapolative generalization to unseen components.

This gap between composition-aware validation and leave-component-out validation is central to the present benchmark. It shows that descriptor-based models may appear useful under interpolation-like conditions while failing to provide reliable predictions for chemically unseen DES components, particularly for transport properties governed by collective and dynamic interactions.

### Baseline and Similarity-Based Benchmarking

To assess whether ML models provide meaningful predictive value beyond simple thermodynamic trends, performance was compared against a set of baseline models, including mean, temperature-only, and composition-based predictors. The results are summarized in Table 4 and Figure 3. The Δ*R* ^2^ values provide a simple effect-size measure of the incremental contribution of the full descriptor-based model beyond trivial and temperature-driven baselines.

**Table 4:** Comparison with Trivial Baselines and Effect-Size Estimates^a^

**Property**	**Model**	***R*** ^**2**^	**Δ*R*** ^**2**^ **vs Dummy**	**Δ*R*** ^**2**^ **vs Temperature**
Density	Dummy	0.000	—	—
Density	Temperature-only	0.320	+0.320	—
Density	Full model	0.857	+0.857	+0.537
Refractive index	Dummy	0.000	—	—
Refractive index	Temperature-only	0.400	+0.400	—
Refractive index	Full model	0.839	+0.839	+0.439
Surface tension	Dummy	0.000	—	—
Surface tension	Temperature-only	0.450	+0.450	—
Surface tension	Full model	0.778	+0.778	+0.328
Conductivity	Dummy	0.000	—	—
Conductivity	Temperature-only	0.500	+0.500	—
Conductivity	Full model	0.535	+0.535	+0.035
Viscosity	Dummy	0.000	—	—
Viscosity	Temperature-only	0.120	+0.120	—
Viscosity	Full model	0.145	+0.145	+0.025

a Δ*R* ^2^ values quantify the incremental predictive gain of the full descriptor-based model relative to dummy and temperature-only baselines.

**Figure 3:**
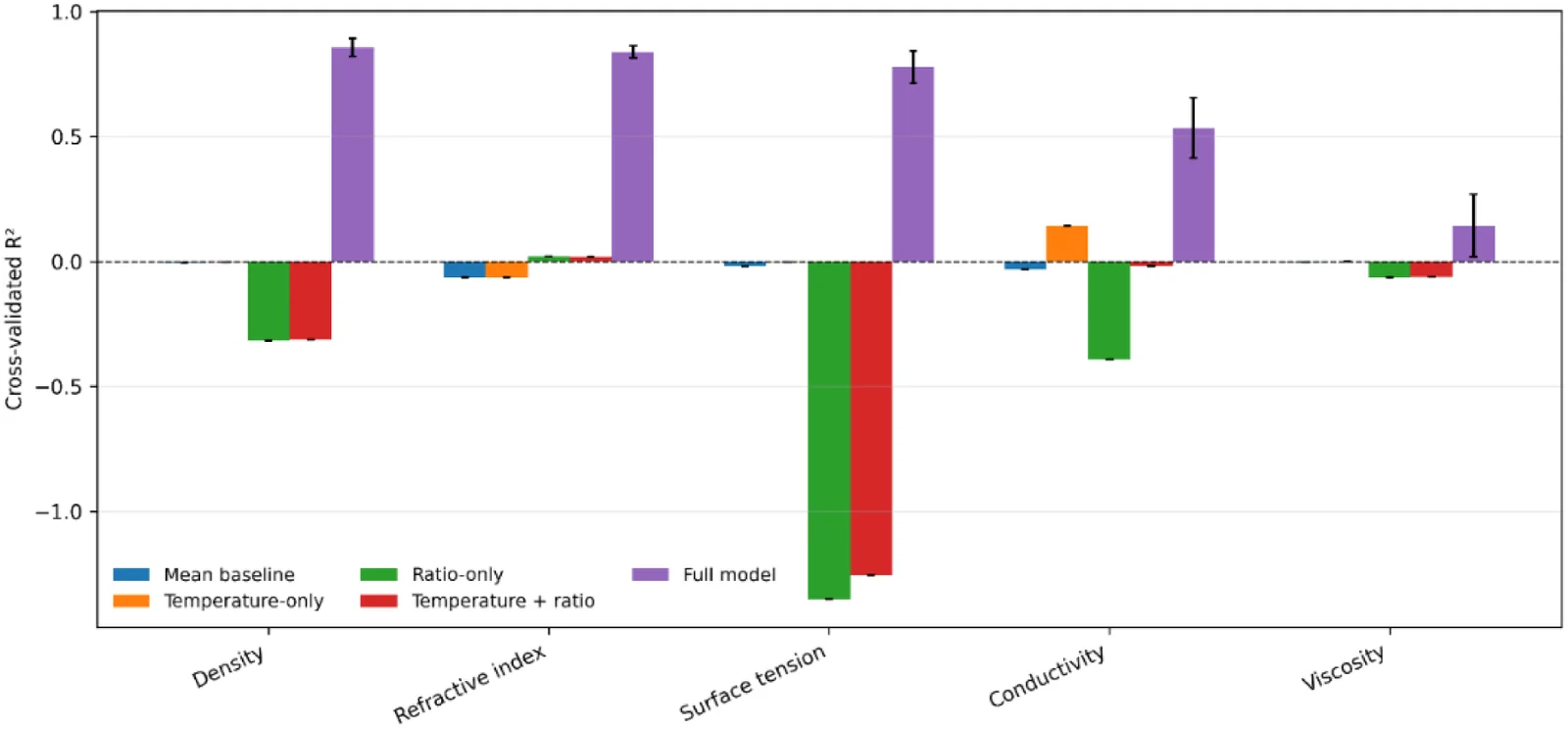
Baseline comparison against the full leakage-safe model.

For density and refractive index, the full models substantially outperform both the mean predictor and the temperature-only baseline, indicating that these properties contain learnable structure–property relationships beyond simple thermodynamic dependencies. This behavior is consistent with prior studies suggesting quasi-additive contributions of molecular components to these properties.^16,17^

Surface tension exhibits intermediate behavior with moderate improvement over temperature-based predictions, reflecting a combined dependence on composition and intermolecular interactions.

In contrast, for electrical conductivity and viscosity, the improvement over temperature-only baseline is limited. In some cases, the full model performs only marginally better than the temperature-only predictor, indicating that the dominant predictive signal arises from thermodynamic variables rather than from descriptor-based features. Similar trends have been reported in early studies of DES conductivity, where strong temperature dependence dominates predictive behavior.^18^

To further examine whether model performance exceeds similarity-based prediction, nearest-neighbor baselines were evaluated under the same validation protocols (Figure S5). Under extrapolative validation (Protocol D), both 1-nearest-neighbor and k-nearest-neighbor models fail to provide meaningful predictions for conductivity and viscosity, with strongly negative *R* ^2^ values and high variance.

This observation indicates that local similarity in the current feature space does not reliably translate to similarity in target properties outside the training domain. For density and refractive index, nearest-neighbor methods exhibit relatively stable, albeit limited, performance, suggesting that local similarity is partially meaningful for these properties.

Combined with the applicability-domain analysis, these results demonstrate that the limitations observed for conductivity and viscosity are not solely due to model choice but are strongly linked to the representational capacity of the descriptor-based feature space. A same-data set comparison across representative model classes further showed that the poor extrapolative behavior of conductivity and viscosity is not unique to the primary ExtraTrees model but persists across linear and nonlinear regressors (Table S10). Because transport properties span broad numerical ranges, additional relative error metrics were calculated for conductivity and viscosity; these metrics confirm the same extrapolative degradation while avoiding overinterpretation of absolute MAE values alone (Table S11). These results indicate that extrapolative prediction in DES systems is constrained primarily by representation rather than the model class. Even similarity-based methods fail under leave-component-out validation, suggesting that feature space proximity does not necessarily correspond to physicochemical similarity for transport properties.

A retrospective recent-literature holdout stress test was further performed by training models on pre-2025 records and evaluating them on 2025–2026 records withheld from training (Table S12). The primary ExtraTrees model retained useful performance for density and refractive index, but transferability deteriorated for surface tension and collapsed for conductivity and viscosity. These results provide additional practical confirmation that the present descriptor-based representation is more reliable for relatively additive properties than for transport properties requiring extrapolation beyond the training domain.

### Feature Contribution and Property-Specific Learnability

The contribution of different feature groups to predictive performance is evaluated through a structured ablation analysis (Figure 4 and Table S2). Across all properties, predictive performance changes with the inclusion of additional feature types; however, the magnitude and origin of this improvement are strongly property-dependent.

**Figure 4:**
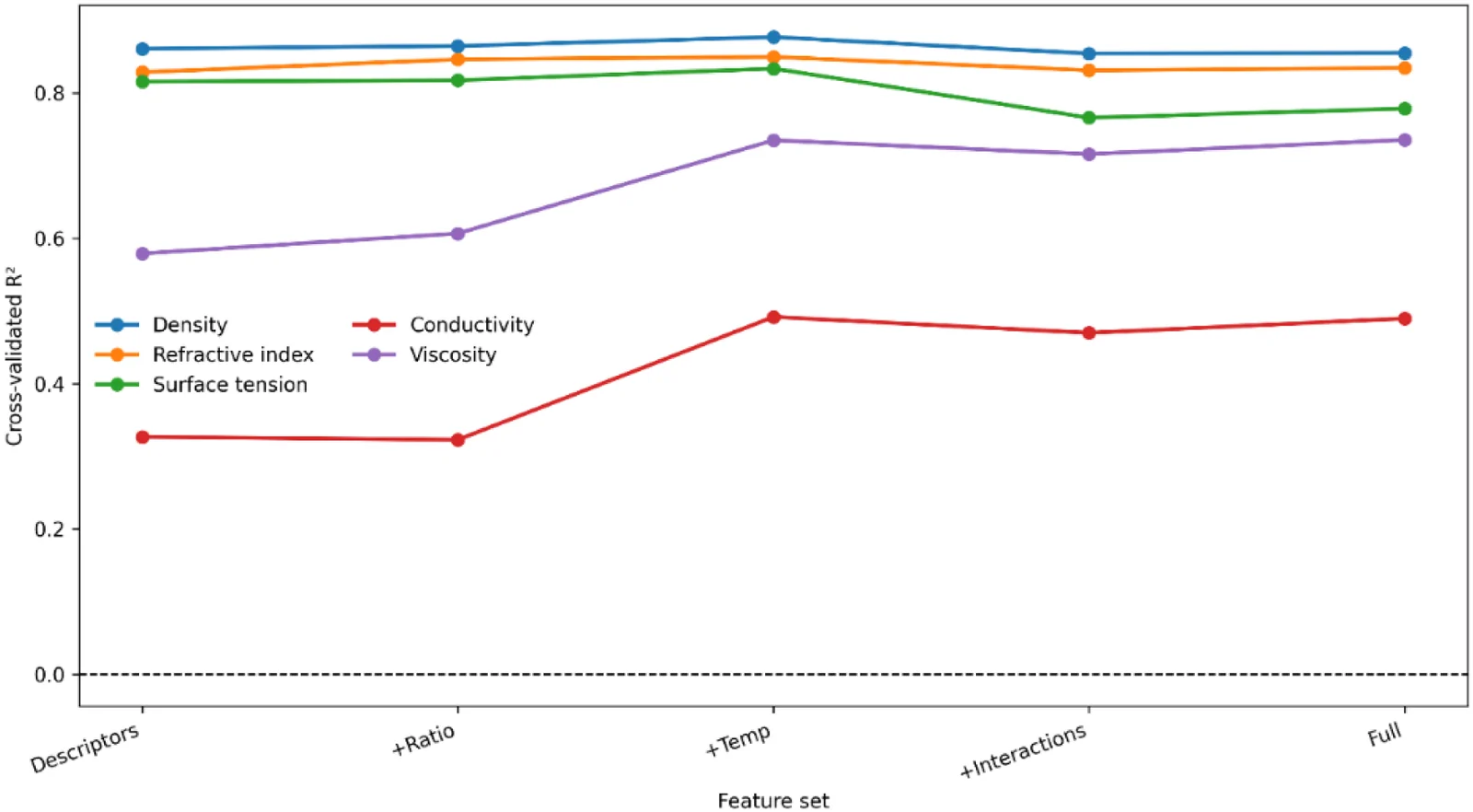
Feature ablation across DES property prediction tasks.

For density and refractive index, molecular descriptors contribute meaningfully to prediction, and further improvements are obtained by incorporating composition and temperature. This pattern indicates the presence of relatively stable and partially transferable structure–property relationships.

Surface tension shows moderate gains from multiple feature groups, reflecting a mixed dependence on the molecular structure, composition, and thermodynamic conditions.

In contrast, for electrical conductivity and viscosity, the dominant improvement arises from the inclusion of temperature, while descriptor-based features provide only a limited additional benefit. Descriptor-only models perform poorly for these properties, and the full feature representation yields only a modest improvement relative to temperature-based baselines.

These results indicate that the effectiveness of descriptor-based representations depends strongly on the underlying physicochemical mechanisms governing the properties. Static molecular descriptors can capture additive or weakly interacting contributions, but they are less effective for properties dominated by collective many-body interactions, such as charge transport and viscous flow.

### Diagnostic Analysis of Viscosity Prediction

Viscosity represents the most challenging target property in the present study and is examined in detail using additional diagnostic analyses (Figure S6). Logarithmic transformation of the target variable improves statistical stability and reduces skewness; however, it does not resolve the limitations in predictive performance under extrapolative validation.

Error analysis reveals persistent and structured deviations across temperature and composition ranges, indicating that key governing mechanisms are not adequately captured by the current feature representation. Most critically, under extrapolative validation (Protocol D), model performance collapses, with *R* ^2^ values approaching zero or becoming negative.

The behavior observed for viscosity cannot be attributed solely to the model choice or target distribution. Rather, it reflects the inability of the current static descriptor space to capture the collective and dynamic interactions governing the viscous behavior in DES systems. This interpretation is consistent with the established understanding that viscosity in hydrogen-bonded liquids is governed by many-body interactions, network formation, and dynamic rearrangements that are not explicitly encoded in static molecular descriptors.^3^

### Applicability-Domain Behavior and Distance-to-Training Effects

To further examine the relationship between model error and chemical-space coverage, an applicability-domain analysis was performed based on feature-space distance to the training data (Figure S4 and Table S6). For each test sample, the prediction error was evaluated as a function of the distance to the nearest training-domain neighborhood.

The distance–error relationship is not strictly monotonic for every property and validation protocol, reflecting the heterogeneity and imbalance of the data set. Nevertheless, several clear patterns emerge. For density, refractive index, and surface tension, errors generally increase between the closest and farthest feature-space quartiles, indicating reduced reliability in sparsely represented regions. For viscosity, large increases in error are observed in several validation settings, especially under leakage-corrected and pair-level validation.

For conductivity, distance-based behavior is more irregular, reflecting the highly heterogeneous distribution and strong temperature dependence of this property. This irregularity is itself informative: feature space proximity under the current static descriptor representation does not consistently capture the physicochemical similarity needed for reliable transport property prediction.

Together with the nearest-neighbor baseline results, the applicability domain analysis shows that extrapolative prediction in DES systems is constrained primarily by representation rather than model class. Even similarity-based methods fail under leave-component-out validation, suggesting that feature space proximity does not necessarily correspond to physicochemical similarity for transport properties.

### Interpretability and Limits of Feature Importance

Model interpretability analysis was performed using permutation feature importance and SHAP methods to identify dominant predictors across properties (Figure S7 and Table S3).

Across all properties, temperature emerges as one of the most influential predictors, followed by selected descriptors associated primarily with HBD components. For density and refractive index, both HBA and HBD descriptors contribute meaningfully to prediction, supporting the existence of underlying structure–property relationships.

For conductivity and viscosity, feature importance is dominated by temperature and a limited subset of descriptors, consistent with baseline and ablation analyses. However, the temperature dominance should be interpreted carefully. Temperature is a physically meaningful variable for thermodynamic and transport properties, but it is also sampled more continuously than chemical composition in the data set and often appears in repeated measurement series for similar DES compositions. As a result, high-temperature importance may partly reflect in-domain measurement structure rather than universally transferable physical control.

Importantly, high feature importance does not necessarily correspond to an extrapolative predictive capability. For example, viscosity models assign importance to temperature and selected HBD descriptors within the training domain yet fail under leave-component-out validation. This observation highlights the distinction between feature relevance within the observed data domain and true generalization beyond it, emphasizing the need for careful interpretation of feature importance in data-driven chemical modeling.^15^

### Practical Implications and Future Representation Strategies

The combined results reveal a clear spectrum of learnability across the DES properties. Density and refractive index exhibit relatively stable and transferable behaviors, indicating that descriptor-based models can capture their dominant mechanisms to a meaningful extent. Surface tension occupies an intermediate regime, with moderate predictability and partial dependence on both structure and thermodynamic variables.

In contrast, electrical conductivity and viscosity show a strong dependence on temperature and a limited contribution from descriptor-based features, with particularly poor extrapolative performance. These findings suggest that the current static descriptor-based representation is limited to properties governed by complex, collective interactions. This limitation is consistent with the known role of hydrogen-bonding networks, microstructure, ion mobility, and dynamic rearrangements in the transport properties of DES systems.^3^

From a practical perspective, data-driven DES design strategies should therefore be tailored to the target property and intended prediction regime. Descriptor-based models may be useful for screening relatively additive or weakly coupled properties, particularly when the candidate DES lies within the domain of previously observed component classes. However, the same representation should not be assumed to provide reliable extrapolation for transport properties or chemically distant systems without additional validation.

The results also indicate concrete directions for improving future DES property models. First, COSMO-RS-derived descriptors, including sigma profiles, sigma moments, and related charge density descriptors, may provide richer information about polarity, hydrogen-bonding capacity, and solvation-relevant molecular environments than static RDKit descriptors alone. Such descriptors have already been used for DES electrical conductivity modeling and related DES thermophysical property prediction.^11,19^ Second, thermodynamics-informed or hybrid descriptors that combine molecular features with physically motivated quantities may improve transferability, particularly when the target property is linked to phase behavior, solvation, or transport mechanisms.^20^

Third, graph-based and mixture-aware representations could explicitly encode the HBA–HBD pair, molar composition, and component interactions rather than treating the system as a concatenation of static component descriptors. Such approaches may be particularly important for ternary DESs, hydrated DESs, and water-containing mixtures, which introduce additional compositional degrees of freedom beyond the binary HBA–HBD representation used in the present benchmark. Finally, simulation-informed descriptors derived from molecular dynamics or related approaches—such as hydrogen-bond lifetimes, radial distribution features, coordination patterns, diffusion coefficients, and structural relaxation metrics—may be required to describe dynamic transport mechanisms more directly.

The present benchmark should therefore be interpreted not as a universal predictive model for all DES formulations but as a leakage-aware assessment of what can and cannot be expected from static descriptor-based representations under increasingly realistic validation conditions. Overall, the results emphasize that model performance in DES systems is strongly conditioned by data set structure, feature representation, and validation design. Future progress will likely require integrating richer interaction-aware descriptors with rigorous validation protocols that distinguish interpolation within known chemical neighborhoods from extrapolation to genuinely unseen DES chemistries.

## Conclusion

In this work, the predictive behavior of descriptor-based ML models for DES properties was systematically evaluated using a curated and high-confidence data set spanning the period 2003–2026. Rather than focusing solely on predictive accuracy, the study examined the reliability, limitations, and transferability of these models under controlled and leakage-aware validation conditions.

The results demonstrate that the apparent model performance is highly sensitive to the validation design. While relatively strong predictive performance can be achieved under leakage-corrected and composition-aware validation, progressively stricter protocols reveal substantial differences between the interpolation-driven accuracy and extrapolative generalization. This effect is consistently observed across all of the investigated properties.

A clear property-dependent behavior emerges from the analysis. Density and refractive index exhibit relatively stable and transferable predictive performance, indicating that descriptor- based models can capture key aspects of their underlying structure–property relationships. Surface tension shows intermediate behavior, with moderate dependence on both structural and thermodynamic variables.

In contrast, electrical conductivity and viscosity display a strong dependence on temperature and a limited contribution from descriptor-based features. In particular, viscosity prediction remains limited under the current data set, static descriptor-based representation, and validation setting. Model performance deteriorates significantly under extrapolative conditions, and both distance-based analysis and similarity-based baselines indicate a strong dependence on proximity to the training domain.

These findings indicate that the primary limitation lies not only in model choice but also in the representational capacity of the feature space. Static descriptor-based representations provide a simplified approximation of DES systems and are insufficient to capture the collective, dynamic interactions governing transport properties.

From a methodological perspective, the results highlight the importance of rigorous evaluation in data-driven chemical modeling. Without explicit control of data leakage and validation design, the model performance may be overestimated and misinterpreted. The present study demonstrates that careful separation of interpolation and extrapolation regimes is essential for assessing the model reliability.

From an application perspective, the results suggest that descriptor-based ML models can be effectively used for screening properties with relatively additive or weakly coupled behavior, such as density and refractive index. However, more expressive representations—potentially incorporating COSMO-RS-derived descriptors, mixture-aware molecular representations, thermodynamics-informed features, or simulation-informed descriptors—are required for accurately modeling transport properties such as viscosity and electrical conductivity under extrapolative conditions.

Overall, this study provides a structured and reproducible assessment of the capabilities and limitations of descriptor-based ML in DES systems and clarifies the conditions under which such models can be expected to provide reliable and transferable predictions.

## Supplementary Material





## Data Availability

GitHub repository and Zenodo archive containing the complete reproducible workflow, frozen GOLD data set, validation pipeline, figure/table generation scripts, and reference outputs associated with this study (DOI: 10.5281/zenodo.21209405).

## Notes

Literature screening, data extraction, and standardization procedures (Sections S1–S2); descriptor construction and feature engineering strategy (Section S3); data set coverage and imbalance analysis (Section S4; Table S4; Figure S1); validation protocols, leakage-audit procedures, model configuration summary, and literature comparison to prior DES ML/QSPR studies (Section S5; Tables S5, S8–S9); baseline models, applicability-domain analysis, nearest-neighbor evaluation, viscosity diagnostics, same-data set model-class benchmarking, transport-property relative-error analysis, recent-literature holdout stress testing, and alternative feature-representation discussion (Section S6; Tables S6–S7, S10–S12; Figures S2–S6); interpretability analysis including SHAP-based feature importance (Section S7; Table S3; Figure S7); code/data availability information and reproducible workflow documentation (Section S8) (PDF)
Reproducible Python workflow containing the frozen GOLD data set, leakage-aware validation pipeline, feature-engineering workflow, baseline and ablation analyses, applicability-domain and nearest-neighbor evaluation scripts, recent-literature holdout and model-class benchmark scripts, main-text figure/table generation scripts, reference outputs, and execution documentation (ZIP)
