# Supplementary material for: A Leakage-Aware Benchmark Study of Machine Learning Models for Deep Eutectic Solvent Property Prediction
Source: ACS Omega. 2026 Aug 7;11(32):48552–62. doi: 10.1021/acsomega.6c05807 (PMC13491872; doi:10.1021/acsomega.6c05807)
Supplement: Supplementary file 2 [file ao6c05807_si_002.zip › Supplementary Information/DES_ML_Audit_Reproducible_Pipeline/reference_outputs/main_text_assets/figures/Figure2_validation_sensitivity.pdf]

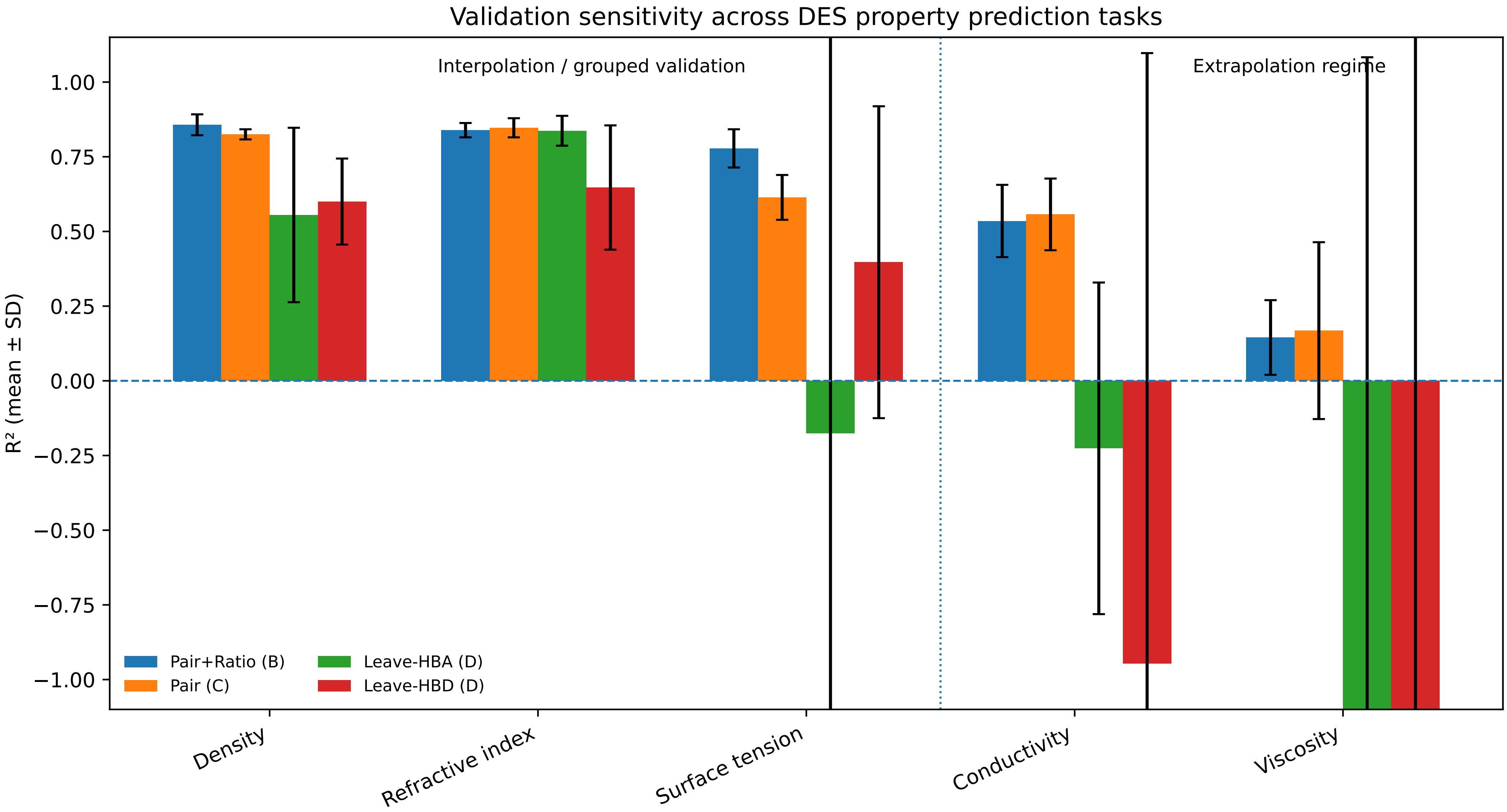

Validation sensitivity across DES property prediction tasks
Interpolation / grouped validation
Extrapolation regime
R² (mean ± SD)
1.00
0.75
0.50
0.25
0.00
−0.25
−0.50
−0.75
−1.00
Pair+Ratio (B)
Pair (C)
Leave-HBA (D)
Leave-HBD (D)
Density
Refractive index
Surface tension
Conductivity
Viscosity
