# Supplementary material for: A Leakage-Aware Benchmark Study of Machine Learning Models for Deep Eutectic Solvent Property Prediction
Source: ACS Omega. 2026 Aug 7;11(32):48552–62. doi: 10.1021/acsomega.6c05807 (PMC13491872; doi:10.1021/acsomega.6c05807)
Supplement: Supplementary file 2 [file ao6c05807_si_002.zip › Supplementary Information/DES_ML_Audit_Reproducible_Pipeline/reference_outputs/main_text_assets/figures/Figure4_feature_ablation.pdf]

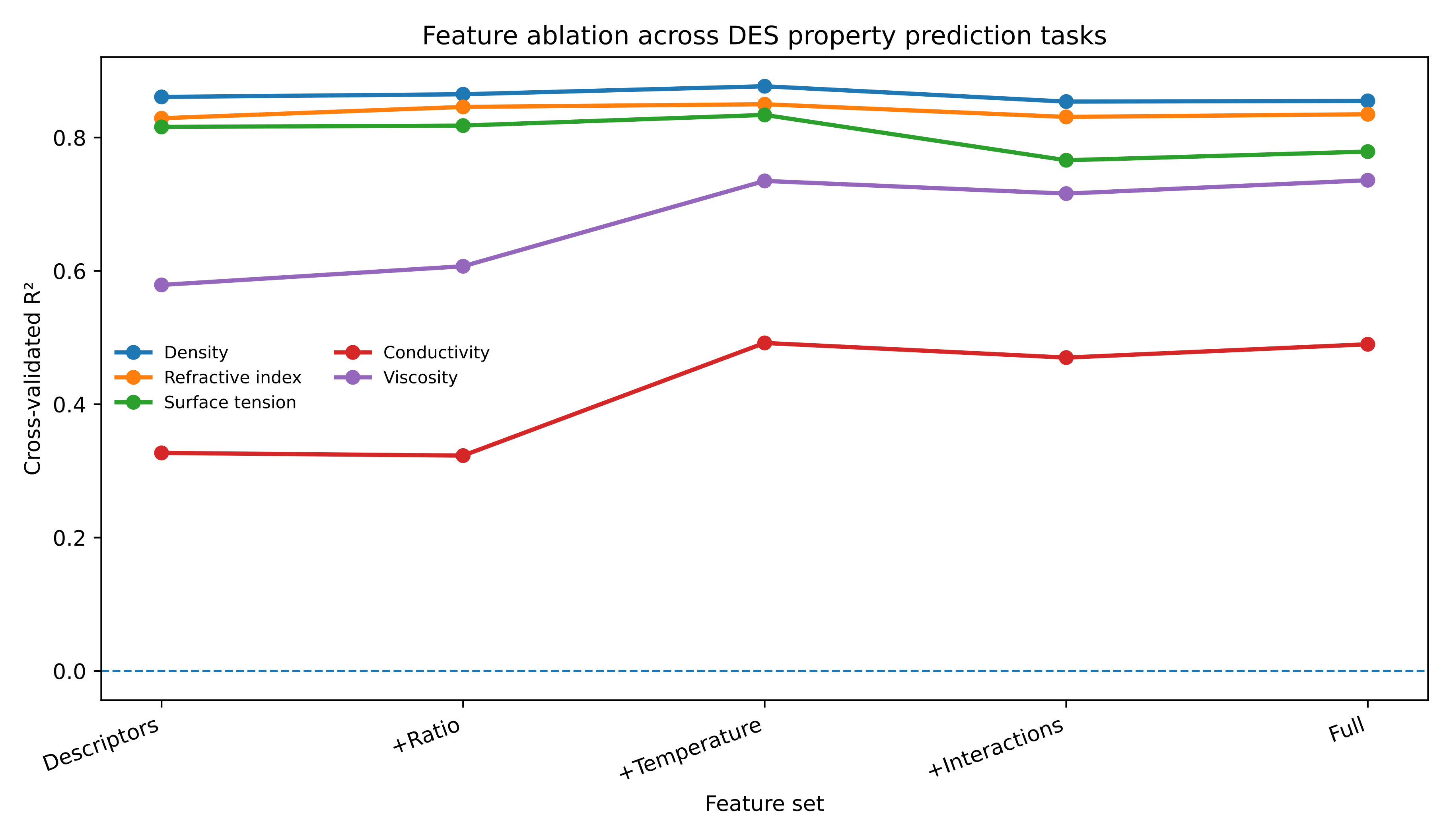
Feature ablation across DES property prediction tasks
Cross-validated R²
0.0
0.2
0.4
0.6
0.8
Density
Refractive index
Surface tension
Conductivity
Viscosity
Descriptors
+Ratio
+Temperature
+Interactions
Full
Feature set
