# Supplementary material for: A Leakage-Aware Benchmark Study of Machine Learning Models for Deep Eutectic Solvent Property Prediction
Source: ACS Omega. 2026 Aug 7;11(32):48552–62. doi: 10.1021/acsomega.6c05807 (PMC13491872; doi:10.1021/acsomega.6c05807)
Supplement: Supplementary file 2 [file ao6c05807_si_002.zip › Supplementary Information/Supplementary Information.pdf]

Supplementary Information

A Leakage-Aware Benchmark Study of Machine Learning Models for Deep Eutectic Solvent Property Prediction

Hakim Faraji [a], *Julio Brito Santana* [b], *Ruth Rodríguez-Ramos* [a, c], *Bárbara Socas-Rodríguez* [a], *Antonio V. Herrera Herrera* [a]

[a] Departamento de Química, Área de Química Analítica, Facultad de Ciencias, Universidad de La Laguna (ULL), Tenerife, Spain

[b] Departamento de Ingeniería Informática y de Sistemas, Universidad de La Laguna (ULL), Tenerife, Spain

[c] CIMO, LA SusTEC, Instituto Politécnico de Bragança, Campus de Santa Apolónia, Bragança, 5300-253, Portugal

S1. Data Sources and Literature Screening

A comprehensive dataset of deep eutectic solvent (DES) physicochemical properties was compiled from published experimental studies spanning the period 2003–2026. The dataset integrates two primary sources. First, a review-based dataset (2003–2021) was constructed from validated literature compilations reporting DES properties. Second, an extended dataset (2021–2026) was assembled through a systematic search of scientific databases, including Scopus and Web of Science, using targeted keywords such as “deep eutectic solvent”, “density”, “viscosity”, “conductivity”, “surface tension”, and “refractive index”.

Publications were screened in two stages. An initial relevance screening identified studies reporting experimentally measured DES properties. This was followed by a quality screening requiring explicit reporting of component identity (HBA and HBD), molar composition, temperature, and property values. Only studies meeting these criteria were included in the curated dataset.

All data were manually extracted to ensure high fidelity and consistency. For each record, the following information was collected: chemical identity of HBA and HBD components, their functional roles, molar ratio, measurement temperature, and the reported physicochemical property. When data were distributed across multiple tables or sections within a publication, cross-verification was performed to ensure internal consistency and to prevent duplication.

To support traceability and reproducibility, extracted records were initially stored in structured intermediate formats (JSON) prior to dataset consolidation. The final dataset was then organized in a structured tabular format, where each row corresponds to a single experimental measurement.

To ensure data quality and enable controlled analysis, a two-tier dataset structure was defined. The GOLD dataset includes only high-confidence records with complete and unambiguous information, clearly defined units, and fully specified experimental conditions. The RELAXED dataset extends this collection by including records with minor incompleteness or inconsistencies that could be reasonably resolved without introducing significant uncertainty. This dual-structure design enables assessment of model sensitivity to data quality.

The final dataset includes standardized variables describing component identity (with corresponding molecular representations such as SMILES), molar composition, temperature (standardized to Kelvin), property type, and property value. Source-level metadata were retained for all records to ensure full traceability and reproducibility of the dataset.

S2. Data Standardization and Integrity Control

To ensure dataset consistency, traceability, and suitability for machine learning applications, a multi-stage data standardization and integrity control pipeline was implemented prior to modeling.

Component normalization and canonical registry. Chemical component names extracted from the literature exhibited significant variability, including synonyms, abbreviations, and inconsistent formatting. To address this, a canonical component registry was constructed. Each unique chemical entity was mapped to a standardized identifier and, where possible, to a corresponding SMILES representation. Manual curation was performed to resolve ambiguities arising from alternative naming conventions (e.g., common names versus IUPAC names) and inconsistent abbreviations. Records with unresolved or ambiguous component identities were excluded from the GOLD dataset.

Duplicate detection and resolution. Duplicate and near-duplicate records were identified using a combination of exact and fuzzy matching criteria based on component identity (HBA/HBD), molar ratio, temperature, and reported property values. When duplicate entries were detected across different sources, preference was given to records with more complete metadata or clearer experimental reporting. Redundant entries were removed to prevent bias in model training and evaluation.

Unit harmonization. All reported property values were converted into a consistent unit system prior to analysis. Temperature was standardized to Kelvin and retained as an explicit modeling variable. Property values were expressed in consistent units, including density (g cm^{-3}), viscosity (mPa s), electrical conductivity (mS cm^{-1}), surface tension (mN m^{-1}), and refractive index (dimensionless). Where necessary, conversion factors were applied based on standard physical relationships. Records with unclear or non-convertible units were excluded from the GOLD dataset but, where appropriate, retained in the RELAXED dataset under controlled assumptions.

Thermodynamic consistency and validation. Each record was evaluated for internal consistency, including verification of temperature ranges, physically plausible property values, and consistency

between reported variables. Outliers and physically implausible entries were flagged and manually reviewed. Records with unresolved inconsistencies were excluded from the high-confidence dataset.

Handling of missing and partially specified data. Records with missing critical variables, such as undefined molar ratio or temperature, were excluded from the GOLD dataset. However, partially specified records with minor omissions were retained in the RELAXED dataset when they could be reasonably interpreted without introducing significant uncertainty.

Dataset tier definition. Based on these criteria, two dataset tiers were defined. The GOLD dataset contains only high-confidence records with complete and internally consistent information, and serves as the basis for all modeling and evaluation reported in the main manuscript. The RELAXED dataset extends this collection by including additional records with minor resolvable inconsistencies, enabling controlled assessment of data-quality sensitivity.

S3. Feature Engineering Framework

To enable robust and descriptor-based representation of DES systems, a multi-level feature engineering strategy was implemented, integrating component-level descriptors, interaction-aware features, composition-dependent encoding, and thermodynamic variables.

Molecular descriptors (component-level). For each DES component (HBA and HBD), molecular descriptors were calculated using RDKit based on canonical SMILES representations derived from the component registry (Section S2). The descriptor set captures structural, physicochemical, and topological properties, including molecular weight, atom counts, heteroatom counts, hydrogen-bond donor and acceptor counts, topological polar surface area (TPSA), logP, molar refractivity, rotatable bonds, ring counts (total, aromatic, and aliphatic), fraction of sp^3 carbons, formal charge, and selected fragment-based indicators.

Pairwise interaction features. To approximate interactions between HBA and HBD species, pairwise features were constructed by combining component-level descriptors. These include arithmetic combinations (e.g., sums, differences, and ratios) as well as interaction proxies intended to capture hydrogen-bonding potential and complementary behavior between components. While these features provide a coarse approximation of intermolecular interactions, they remain simplified representations of inherently complex many-body effects.

Composition-aware encoding. Molar ratios were explicitly incorporated into the feature space to reflect the compositional dependence of DES properties. Ratio-aware features were constructed by weighting component descriptors according to their molar fractions and by including explicit HBA/HBD ratio variables. This representation enables differentiation between systems with identical components but different compositions.

Temperature handling. Temperature was included as an explicit numerical feature for all records and standardized to Kelvin (Section S2). No additional transformation was applied at this stage, allowing models to directly learn temperature-dependent trends.

Feature assembly and preprocessing. All features were combined into a unified feature matrix. Basic preprocessing steps were applied, including removal of constant or near-constant features and optional scaling for models requiring normalized inputs. The feature space was constructed consistently across all property-specific datasets.

To ensure leakage-safe modeling, all target variables and non-target property columns were explicitly removed from the feature matrix prior to training under leakage-corrected validation protocols (Section S5). The final representation thus integrates molecular, compositional, and thermodynamic information while maintaining strict separation between input features and prediction targets.

S4. Dataset Structure, Coverage, and Imbalance Analysis

Property-specific coverage is summarized in Table S1, and extended imbalance statistics are reported in Table S4. Extended dataset-structure analyses are provided here to support the summary presented in the main manuscript. The curated GOLD dataset was partitioned into property-specific subsets corresponding to independent regression tasks, including density, viscosity, electrical conductivity, surface tension, and refractive index. Only records with complete and valid measurements for each property were retained.

To characterize dataset structure and potential sources of bias, quantitative analyses of component frequency, compositional coverage, and thermodynamic distribution were performed.

Component frequency distributions reveal a pronounced long-tail behavior for both hydrogen bond acceptor (HBA) and hydrogen bond donor (HBD) species (Figure S1). A relatively small subset of components accounts for a large fraction of the dataset, while many components are represented by only a limited number of records. This imbalance is further quantified using summary statistics, including top-component contribution and inequality metrics (Table S4).

Molar ratio distribution is similarly non-uniform (Figure S1), with a strong concentration around commonly reported compositions (typically between 1 and 3), and sparse coverage of more extreme ratios. This uneven compositional coverage limits the representation of the full design space and may bias model behavior toward densely sampled regions.

Temperature distribution across properties (Figure S1) shows relatively consistent coverage, but with a clear concentration around near-ambient condition. Such bias is particularly relevant for temperature-sensitive properties, including viscosity and electrical conductivity.

Together, these results indicate that the dataset is dominated by densely sampled regions of chemical and thermodynamic space, with limited representation of rare components and extreme conditions. This imbalance has direct implications for model evaluation, as models may exploit repeated chemical patterns under random or weakly constrained splits, leading to optimistic performance estimates.

These observations motivate the use of group-aware and extrapolative validation protocols in the main analysis to ensure a more realistic assessment of model generalization.

S5. Modeling Framework, Validation Protocols, and Leakage Audit

Model selection. Three regression model classes were employed to provide a balanced representation of linear and non-linear learning:

- **Ridge regression**, representing a linear baseline with L2 regularization
- **ExtraTrees regressor**, serving as the primary non-linear ensemble model
- **HistGradientBoosting regressor**, included as a gradient boosting benchmark

These models were selected to cover a range of model complexities while maintaining interpretability and minimizing the risk of overfitting associated with highly complex architectures.

Hyperparameter configuration. Model hyperparameters were specified using fixed, default-informed configurations selected before final evaluation. They were not optimized on the test folds, in order to avoid overfitting validation protocols and to maintain comparability across properties and validation settings. Model hyperparameters were specified using fixed, default-informed configurations selected before final evaluation. They were not optimized on the test folds, in order to avoid overfitting validation protocols and to maintain comparability across properties and validation settings. Stage-specific configurations are summarized in Table S8.

To ensure fair comparison, all models were trained and evaluated under identical data splits for each validation protocol.

Model configurations and preprocessing. To improve reproducibility, the principal model configurations used across the workflow are summarized in Table S8. The configurations were fixed before final evaluation and were not optimized separately on the test folds. Diagnostic, extrapolative, feature-ablation, applicability-domain, and interpretability analyses used stage-specific configurations

to balance computational efficiency with reproducibility. All preprocessing operations, including imputation and scaling where applicable, were fitted within each training fold only.

Validation protocols. To systematically evaluate model reliability and generalization, four validation protocols were defined:

Protocol A was used exclusively for diagnostic purposes and was not considered a valid performance estimate. Protocols B–D were leakage-safe and form the basis for all conclusions reported in the main manuscript.

Group-aware and extrapolative data splitting. To prevent data leakage, grouping strategies were applied during data splitting. In Protocol B, samples were grouped based on the combined identity of HBA, HBD, and molar ratio, ensuring that repeated measurements of the same DES composition were not split across training and testing sets.

In Protocol C, grouping was performed at the HBA–HBD pair level, excluding composition-level overlap between training and test sets. In Protocol D, leave-component-out strategies were applied, where either HBA or HBD species were completely excluded from the training set and used only for testing. This enables direct evaluation of extrapolative generalization.

Leakage audit. A formal leakage audit was performed for all modeling workflows. In leakage-safe protocols, explicit checks were applied to ensure that:

- the target variable was not present in the feature matrix
- no other property columns were included as features
- feature construction did not implicitly encode target information

Leakage checks were applied consistently across full models, baseline models, and feature-ablation experiments. This ensured that all reported performance reflects genuine predictive learning rather than information leakage.

Multi-seed evaluation. To account for variability introduced by data splitting, model training and evaluation were repeated across multiple random seeds. Performance metrics were averaged across runs to obtain stable estimates.

Evaluation metrics. Model performance was evaluated using:

- coefficient of determination (R^2)
- mean absolute error (MAE)

These metrics provide complementary measures of predictive accuracy and robustness across different properties and validation protocols. For summary performance tables, 95% confidence intervals were

calculated as mean ± 1.96 × SD/√n, where n denotes the number of repeated validation folds/runs used to estimate the reported mean and standard deviation.

Contextual comparison to prior DES ML/QSPR studies. To contextualize the present benchmark relative to prior DES property-prediction studies, representative DES ML/QSPR and mixture-QSPR studies are summarized in Table S9. The comparison highlights that prior studies have advanced property-specific prediction, online DES property platforms, COSMO-RS-based descriptor strategies, thermodynamics-informed models, and mixture-QSPR validation concepts. The present work differs by focusing on leakage-aware validation, applicability-domain behavior, leave-component-out extrapolation, and reproducible benchmarking across multiple DES properties.

S6. Baseline, Applicability Domain, and Diagnostic Analyses

S6.1. Baseline models

The performance gain relative to the temperature-only baseline is further summarized in Figure S2. Baseline models were evaluated to determine whether machine learning models provide predictive value beyond simple thermodynamic and compositional predictors. The evaluated baselines include mean prediction, temperature-only, ratio-only, and combined temperature–ratio models.

The incremental contribution of the full descriptor–ratio–temperature representation relative to descriptor-only models is shown in Figure S3. Results are summarized in the main manuscript (Table 4 and Figure 3), while extended outputs are provided in this section.

S6.2. Applicability-domain analysis

To assess the relationship between prediction error and proximity to the training data, an applicability-domain analysis was performed using feature-space distance metrics.

For each test sample, the distance to the k nearest training samples was computed, and prediction error was analyzed as a function of this distance. Samples were grouped into quartiles based on distance to enable comparison across different regions of feature space.

The results (Figure S4) show that prediction error generally increases with increasing distance from the training domain. This trend is weak for density and refractive index, moderate for surface tension, and pronounced for electrical conductivity and viscosity.

These findings indicate that model performance is strongly influenced by proximity to previously observed data and that extrapolative predictions are associated with increased uncertainty.

S6.3. Nearest-neighbor baseline

To further evaluate whether model performance exceeds similarity-based prediction, nearest-neighbor (NN) models were evaluated under the same validation protocols.

Under extrapolative validation (leave-component-out), both 1-NN and k-NN regressors show poor performance for conductivity and viscosity, with strongly negative R^2 values and high variance (Figure S5). This indicates that local similarity in the current feature space is not sufficient to support reliable prediction outside the training domain.

For density and refractive index, NN models exhibit relatively stable performance, suggesting that local similarity is partially informative for these properties.

S6.4. Viscosity diagnostics

Additional analyses were performed to investigate the behavior of viscosity prediction, including target transformation and residual analysis. Extended viscosity diagnostics are shown in Figure S6.

Logarithmic transformation improves statistical stability but does not resolve the fundamental limitations under extrapolative validation. Error distributions remain structured and increase significantly for samples located in sparsely represented regions of feature space.

These results confirm that viscosity prediction is limited by the representational capacity of the current descriptor-based feature space rather than by model choice alone.

S6.5. Same-dataset model-class benchmark

To evaluate whether the observed trends were specific to the primary ExtraTrees model, a same-dataset model-class benchmark was performed using representative regression models trained and evaluated on the frozen GOLD dataset under leakage-safe grouped validation. The evaluated models included Ridge regression, RandomForest regression, ExtraTrees regression, and HistGradientBoosting regression where computationally feasible.

The benchmark was designed to compare model classes under identical feature representations and validation protocols rather than to perform extensive hyperparameter optimization. All models used the same leakage-safe descriptor–ratio–temperature feature space, and preprocessing was fitted within each training fold.

The results (Table S10) show that non-linear tree-based models generally outperform the linear Ridge baseline for density, refractive index, and surface tension. However, the degradation observed for conductivity and viscosity under extrapolative validation persists across model classes. These findings support the conclusion that the major limitations for transport-property prediction arise primarily from the current static descriptor representation and dataset coverage rather than from the choice of a single model architecture.

S6.6. Transport-property relative-error metrics

Because transport properties span broad numerical ranges, absolute-error metrics such as MAE and RMSE may not fully reflect the practical magnitude of prediction errors, particularly for viscosity and electrical conductivity. Therefore, relative-error metrics were additionally calculated for the primary ExtraTrees model under internal leakage-safe validation and the recent-literature holdout stress test (Table S11).

For electrical conductivity, relative-error metrics are especially sensitive to very small experimental target values. As a result, MAPE can become extremely large and should not be interpreted alone. For example, conductivity shows high MAPE values under both internal validation and the recent-literature holdout setting, while MdAPE provides a more robust summary of typical relative error. For this reason, MdAPE is reported alongside MAPE throughout Table S11.

For viscosity, relative-error metrics confirm the same conclusion obtained from R^2, MAE, RMSE, applicability-domain analysis, and nearest-neighbor benchmarking. Although interpolation-like validation produces lower relative errors than extrapolative validation, errors increase substantially under leave-component-out and recent-literature holdout conditions. In particular, the recent-literature holdout stress test gives very large relative errors for viscosity, confirming poor transferability beyond the training domain.

Overall, the transport-property relative-error analysis reinforces the conclusion that conductivity and viscosity remain difficult to predict under the current dataset, static descriptor representation, and validation setting. These results support the need for richer interaction-aware or physics-informed representations when modeling DES transport properties.

S6.7. Recent-literature holdout stress test

To evaluate practical predictive behavior beyond the internal validation protocols, a retrospective recent-literature holdout stress test was performed. Records published in 2025–2026 were excluded from model training and used only as a holdout test set, whereas records published before 2025 were used for training. This analysis should be interpreted as an independent recent-literature stress test based on the curated GOLD corpus, not as newly generated experimental validation.

The holdout set contained 619 records across the five modeled properties. ExtraTrees models trained on the pre-2025 training pool retained useful predictive performance for density and refractive index, with R^2 values of 0.669 and 0.823, respectively (Table S12). In contrast, surface tension showed weak transferability, and conductivity and viscosity displayed severe degradation under the recent-literature holdout setting.

These results reinforce the main conclusion that descriptor-based representations are more reliable for relatively additive properties, whereas transport properties remain difficult to extrapolate beyond the training domain. The large relative errors observed for conductivity and viscosity further support the need for more expressive, interaction-aware representations for transport-property prediction.

S6.8. Implications for alternative feature representations

The combined validation, applicability-domain, nearest-neighbor, same-dataset benchmark, and recent-literature holdout analyses indicate that transport-property prediction is limited by the current static descriptor representation. Future feature spaces may benefit from COSMO-RS-derived descriptors, including sigma profiles and related charge-density descriptors, which can encode polarity and hydrogen-bonding environments more directly than simple molecular descriptors. Thermodynamics-informed descriptors and hybrid data-driven representations may further improve transferability for properties linked to solvation or phase behavior.

For viscosity and conductivity, simulation-informed descriptors derived from molecular dynamics may be particularly valuable. Examples include hydrogen-bond lifetimes, radial distribution features, coordination numbers, diffusion coefficients, ion-mobility descriptors, and structural relaxation metrics. Such descriptors could provide more direct access to the collective and dynamic mechanisms that govern transport behavior in DES systems.

Finally, graph-based and mixture-aware representations may be required for modeling ternary DESs, hydrated DESs, and water-containing mixtures. These systems introduce additional compositional degrees of freedom and should not be assumed to fall within the validated domain of binary HBA–HBD descriptor models.

S7. Interpretability Analysis

Permutation importance and SHAP were used to evaluate the dominant predictors in leakage-safe models. Interpretability analysis was treated as supportive evidence and interpreted together with validation, baseline, and ablation analyses.

For the ExtraTrees model under leakage-corrected pair+ratio validation, the highest-ranking SHAP features were dominated by temperature and selected HBD descriptors, depending on the property. The top SHAP-ranked features are summarized in Table S3, and the corresponding property-wise SHAP summary is shown in Figure S7.

The interpretability results support the main conclusion that descriptor relevance is property-dependent. However, feature importance alone does not demonstrate extrapolative transferability; this distinction is particularly important for viscosity, where important features are identified within the training domain but leave-component-out prediction fails.

S8. Code and Data Availability

The curated GOLD dataset used in this study is provided as part of the Supplementary Information and is also available in a structured, machine-readable format in the accompanying data repository.

All scripts used for data processing, feature engineering, model training, validation auditing, baseline analysis, feature ablation, and figure/table generation are included in the associated code repository. The workflow is organized as a reproducible pipeline, enabling regeneration of all main-text tables and figures from the dataset.

For computational efficiency, the pipeline supports a lightweight execution mode (--fast) that reproduces all main manuscript results, as well as an extended mode for generating supplementary analyses.

A frozen version of the code and dataset corresponding to this manuscript is archived at:
https://doi.org/10.5281/zenodo.21209405

References

(1) Halder, A. K.; Haghbakhsh, R.; Voroshylova, I. V; Duarte, A. R. C.; Cordeiro, M. N. D. S. Predicting the Surface Tension of Deep Eutectic Solvents: A Step Forward in the Use of Greener Solvents. *Molecules* **2022**, *27* (15). https://doi.org/10.3390/molecules27154896.

(2) Odegova, V.; Lavrinenko, A.; Rakhmanov, T.; Sysuev, G.; Dmitrenko, A.; Vinogradov, V. DESignSolvents: An Open Platform for the Search and Prediction of the Physicochemical Properties of Deep Eutectic Solvents. *Green Chemistry* **2024**, *26* (7), 3958–3967. https://doi.org/10.1039/d3gc04533a.

(3) Lemaoui, T.; Boublia, A.; Darwish, A. S.; Alam, M.; Park, S.; Jeon, B.-H.; Banat, F.; Benguerba, Y.; AlNashef, I. M. Predicting the Surface Tension of Deep Eutectic Solvents Using Artificial Neural Networks. *ACS Omega* **2022**, *7* (36), 32194–32207. https://doi.org/10.1021/acsomega.2c03458.

(4) Lemaoui, T.; Darwish, A. S.; Hammoudi, N. E. H.; Abu Hatab, F.; Attoui, A.; Alnashef, I. M.; Benguerba, Y. Prediction of Electrical Conductivity of Deep Eutectic Solvents Using COSMO-RS Sigma Profiles as Molecular Descriptors: A Quantitative Structure–Property Relationship Study. *Ind. Eng. Chem. Res.* **2020**, *59* (29), 13343–13354. https://doi.org/10.1021/acs.iecr.0c02542.

(5) Halder, A. K.; Haghbakhsh, R.; Ferreira, E. S. C.; Duarte, A. R. C.; Cordeiro, M. N. D. S. Machine Learning-Driven Prediction of Deep Eutectic Solvents' Heat Capacity for Sustainable Process Design. *J. Mol. Liq.* **2025**, *418*, 126707. https://doi.org/10.1016/j.molliq.2024.126707.

(6) Fu, Y.; Mu, W.; Jovein, I. B.; Chen, B.; Held, C.; Yu, G. Hybrid Thermodynamics-Informed Data-Driven Models for Predicting Fluorinated Gas Solubility in Deep Eutectic Solvents. *AIChE Journal* **2026**, *72* (6), e70299. https://doi.org/10.1002/aic.70299.

(7) Oprisiu, I.; Novotarskyi, S.; Tetko, I. V. Modeling of Non-Additive Mixture Properties Using the Online CHEmical Database and Modeling Environment (OCHEM). *J. Cheminform.* **2013**, *5* (1), 4. https://doi.org/10.1186/1758-2946-5-4.

Table S1. Property-specific coverage of the frozen GOLD dataset.

Property	Target column	N	Unique HBA	Unique HBD	Unique HBA–HBD pairs	Temperature range (K)	Target range
Density	density_g_cm3	1024	79	97	223	273.15–363.15	0.829–1.677
Viscosity	viscosity_mpa_s	944	94	104	257	273.15–368.15	4.106–330000
Conductivity	conductivity_ms_cm	545	44	55	128	278.15–343.15	0.00139–4683
Surface tension	surface_tension_mn_m	469	36	47	90	288.15–353.15	20.2–75.6
Refractive index	refractive_index	511	39	57	119	288.15–353.00	1.426–1.568

Sample size, raw component-label diversity, HBA–HBD pair coverage, temperature range, and target range for each property-specific modeling task.

Table S2. Quantitative feature ablation results under leakage-corrected pair+ratio validation.

Property	Target variant	Descriptors	+ Ratio	+ Temperature	+ Interactions	Full
Density	raw	0.861	0.865	0.877	0.854	0.855
Refractive index	raw	0.829	0.846	0.850	0.831	0.835
Surface tension	raw	0.816	0.818	0.834	0.766	0.779
Conductivity	raw	0.327	0.323	0.492	0.470	0.490
Viscosity	log10	0.579	0.607	0.735	0.716	0.736

Table S3. SHAP-based top features under leakage-corrected pair+ratio validation.

Property	Rank	Feature	Feature label	Mean |SHAP|
Density	1	hbd_descriptor_hetero_atom_count	HBD hetero atom count	0.0167
Density	2	hbd_descriptor_rotatable_bonds	HBD rotatable bonds	0.0119
Density	3	hbd_descriptor_logp	HBD logP	0.0110
Density	4	hba_descriptor_contains_metal_like_atom	HBA contains metal like atom	0.0094
Density	5	hbd_descriptor_tpsa	HBD TPSA	0.0092
Density	6	hba_descriptor_logp	HBA logP	0.0091
Density	7	hbd_descriptor_fraction_csp3	HBD fraction Csp3	0.0072
Density	8	hbd_descriptor_hbond_donors	HBD H-bond donors	0.0064
Refractive index	1	hbd_descriptor_aromatic_ring_count	HBD aromatic ring count	0.0120
Refractive index	2	hbd_descriptor_ring_count	HBD ring count	0.0084
Refractive index	3	hbd_descriptor_fraction_csp3	HBD fraction Csp3	0.0069
Refractive index	4	hba_descriptor_qed	HBA QED	0.0031
Refractive index	5	hba_descriptor_ring_count	HBA ring count	0.0028
Refractive index	6	hba_descriptor_aromatic_ring_count	HBA aromatic ring count	0.0025
Refractive index	7	hbd_descriptor_hbond_acceptors	HBD H-bond acceptors	0.0018
Refractive index	8	hba_descriptor_fraction_csp3	HBA fraction Csp3	0.0018
Surface tension	1	hbd_descriptor_logp	HBD logP	1.235
Surface tension	2	hbd_descriptor_hbond_acceptors	HBD H-bond acceptors	1.204
Surface tension	3	hbd_descriptor_fraction_csp3	HBD fraction Csp3	1.175
Surface tension	4	hba_descriptor_atom_count_total	HBA atom count total	0.9723
Surface tension	5	hba_descriptor_logp	HBA logP	0.8343
Surface tension	6	hba_descriptor_heavy_atom_count	HBA heavy atom count	0.8243
Surface tension	7	hbd_descriptor_rotatable_bonds	HBD rotatable bonds	0.7782
Surface tension	8	hba_descriptor_molar_refractivity	HBA molar refractivity	0.6787
Conductivity	1	temperature_k	Temperature	92.95
Conductivity	2	measurement_temperature_c	Temperature	89.36
Conductivity	3	hba_descriptor_qed	HBA QED	67.51
Conductivity	4	hbd_descriptor_qed	HBD QED	48.28
Conductivity	5	hba_descriptor_fraction_csp3	HBA fraction Csp3	33.59
Conductivity	6	hbd_descriptor_hbond_donors	HBD H-bond donors	28.75
Conductivity	7	hba_descriptor_aromatic_ring_count	HBA aromatic ring count	28.17
Conductivity	8	hba_descriptor_ring_count	HBA ring count	26.00
Viscosity	1	measurement_temperature_c	Temperature	0.1350
Viscosity	2	temperature_k	Temperature	0.1339
Viscosity	3	hbd_descriptor_hbond_donors	HBD H-bond donors	0.1197
Viscosity	4	hbd_descriptor_fraction_csp3	HBD fraction Csp3	0.1163
Viscosity	5	molar_ratio_numeric	Molar ratio	0.0674
Viscosity	6	hbd_descriptor_hbond_acceptors	HBD H-bond acceptors	0.0560
Viscosity	7	hbd_descriptor_tpsa	HBD TPSA	0.0546
Viscosity	8	hbd_descriptor_hetero_atom_count	HBD hetero atom count	0.0545

Table S4. Dataset imbalance summary for GOLD property-specific records. Statistical measures of component frequency, compositional coverage, and thermodynamic distribution, including top-component contribution, singleton counts, and Gini coefficients.

Property	N	Temperature range (K)	Median T (K)	Ratio range	Median ratio	Unique HBA–HBD pairs	HBA unique	HBA top-1 share (%)	HBA top-5 share (%)	HBA top-10 share (%)	HBA singleton count	HBA Gini	HBA most common (count)	HBD unique	HBD top-1 share (%)	HBD top-5 share (%)	HBD top-10 share (%)	HBD singleton count	HBD Gini	HBD most common (count)
Overall_property_rows	3493	273.15–368.15	313.15	0.09–20.00	2.000	302	99	21.1	47.2	62.5	4	0.755	Benzyl triethylammonium chloride (737)	122	9.3	38.6	57.5	20	0.748	phenol (326)
Density	1024	273.15–363.15	313.15	0.09–20.00	2.000	223	79	20.0	48.7	63.1	21	0.708	Choline chloride (205)	97	14.1	43.6	60.9	30	0.698	Glycerol (144)
Viscosity	944	273.15–368.15	313.15	0.09–20.00	2.000	257	94	20.2	47.1	60.2	25	0.697	Choline chloride (191)	104	12.7	41.2	59.3	39	0.693	Levulinic acid (120)
Conductivity	545	278.15–343.15	313.15	0.09–20.00	2.000	128	44	26.2	54.9	73.2	12	0.658	Benzyl triethylammonium chloride (143)	55	12.1	41.7	61.8	15	0.610	phenol (66)
Surface tension	469	288.15–353.15	313.15	0.09–20.00	2.000	90	36	32.8	58.6	78.7	10	0.669	Benzyl triethylammonium chloride (154)	47	14.1	42.4	63.1	13	0.587	phenol (66)
Refractive index	511	288.15–353.00	313.15	0.09–20.00	2.000	119	39	28.0	58.7	77.3	9	0.671	Benzyl triethylammonium chloride (143)	57	12.9	41.7	59.5	16	0.606	phenol (66)

Table S5. Leakage-audit summary across feature sets and validation protocols. Verification of leakage-safe conditions, including absence of target variables and other property columns in the feature matrix, grouping definitions, and audit status across all workflows.

**Workflow / feature set**	**Protocol**	**Grouping definition**	**Targets audited**	**Feature count range**	**Target in X**	**Other property columns in X**	**Group count range**	**Audit status**
Descriptors + ratio	B_pair_ratio	HBA–HBD–ratio grouping	5	53–53	No	No	126–350	PASS
Descriptors + ratio	C_pair	HBA–HBD pair grouping	5	53–53	No	No	86–250	PASS
Descriptors + ratio	D_leave_HBA	Leave-HBA-out grouping	5	53–53	No	No	28–82	PASS
Descriptors + ratio	D_leave_HBD	Leave-HBD-out grouping	5	53–53	No	No	41–94	PASS
Descriptors + temperature	B_pair_ratio	HBA–HBD–ratio grouping	5	53–53	No	No	126–350	PASS
Descriptors + temperature	C_pair	HBA–HBD pair grouping	5	53–53	No	No	86–250	PASS
Descriptors + temperature	D_leave_HBA	Leave-HBA-out grouping	5	53–53	No	No	28–82	PASS
Descriptors + temperature	D_leave_HBD	Leave-HBD-out grouping	5	53–53	No	No	41–94	PASS
Descriptors only	B_pair_ratio	HBA–HBD–ratio grouping	5	51–51	No	No	126–350	PASS
Descriptors only	C_pair	HBA–HBD pair grouping	5	51–51	No	No	86–250	PASS
Descriptors only	D_leave_HBA	Leave-HBA-out grouping	5	51–51	No	No	28–82	PASS
Descriptors only	D_leave_HBD	Leave-HBD-out grouping	5	51–51	No	No	41–94	PASS
Full descriptor + ratio + temperature	B_pair_ratio	HBA–HBD–ratio grouping	5	55–55	No	No	126–350	PASS
Full descriptor + ratio + temperature	C_pair	HBA–HBD pair grouping	5	55–55	No	No	86–250	PASS
Full descriptor + ratio + temperature	D_leave_HBA	Leave-HBA-out grouping	5	55–55	No	No	28–82	PASS
Full descriptor + ratio + temperature	D_leave_HBD	Leave-HBD-out grouping	5	55–55	No	No	41–94	PASS
Ratio-only baseline	B_pair_ratio	HBA–HBD–ratio grouping	5	3–3	No	No	126–350	PASS
Ratio-only baseline	C_pair	HBA–HBD pair grouping	5	3–3	No	No	86–250	PASS
Ratio-only baseline	D_leave_HBA	Leave-HBA-out grouping	5	3–3	No	No	28–82	PASS
Ratio-only baseline	D_leave_HBD	Leave-HBD-out grouping	5	3–3	No	No	41–94	PASS
Temperature + ratio baseline	B_pair_ratio	HBA–HBD–ratio grouping	5	5–5	No	No	126–350	PASS
Temperature + ratio baseline	C_pair	HBA–HBD pair grouping	5	5–5	No	No	86–250	PASS
Temperature + ratio baseline	D_leave_HBA	Leave-HBA-out grouping	5	5–5	No	No	28–82	PASS
Temperature + ratio baseline	D_leave_HBD	Leave-HBD-out grouping	5	5–5	No	No	41–94	PASS
Temperature-only baseline	B_pair_ratio	HBA–HBD–ratio grouping	5	2–2	No	No	126–350	PASS
Temperature-only baseline	C_pair	HBA–HBD pair grouping	5	2–2	No	No	86–250	PASS
Temperature-only baseline	D_leave_HBA	Leave-HBA-out grouping	5	2–2	No	No	28–82	PASS
Temperature-only baseline	D_leave_HBD	Leave-HBD-out grouping	5	2–2	No	No	41–94	PASS

Table S6. Applicability-domain error summary based on k-nearest-neighbor distance.

Property	Protocol	MAE Q1	MAE Q4	Q4/Q1 MAE
Conductivity	B: pair+ratio	6.368	195.040	30.630
Conductivity	C: pair	381.640	115.080	0.302
Conductivity	D: leave-HBA-out	121.410	375.610	3.094
Conductivity	D: leave-HBD-out	442.690	170.630	0.385
Density	B: pair+ratio	0.0081	0.0395	4.896
Density	C: pair	0.0195	0.0389	2.000
Density	D: leave-HBA-out	0.0347	0.0795	2.291
Density	D: leave-HBD-out	0.0243	0.0734	3.014
Refractive index	B: pair+ratio	0.0058	0.0118	2.023
Refractive index	C: pair	0.0090	0.0093	1.035
Refractive index	D: leave-HBA-out	0.0083	0.0153	1.845
Refractive index	D: leave-HBD-out	0.0075	0.0144	1.929
Surface tension	B: pair+ratio	1.522	4.868	3.198
Surface tension	C: pair	2.730	5.126	1.878
Surface tension	D: leave-HBA-out	5.199	6.896	1.326
Surface tension	D: leave-HBD-out	3.680	5.988	1.627
Viscosity	B: pair+ratio	109.870	2390.300	21.760
Viscosity	C: pair	69.900	1895.400	27.120
Viscosity	D: leave-HBA-out	2251.000	1684.200	0.748
Viscosity	D: leave-HBD-out	157.540	1677.300	10.650

Only the closest and farthest feature-space quartiles are shown. Q1 corresponds to test samples closest to the training domain, whereas Q4 corresponds to the farthest samples. Full quartile-level outputs are provided in the data repository.

Table S7. Simplified nearest-neighbor baseline comparison under interpolation-like and extrapolative validation.

Property	Protocol	Model	R^2	MAE
Conductivity	B_pair_ratio	Dummy mean	-0.0162 ± 0.0192	412.92 ± 73.11
Conductivity	B_pair_ratio	1-NN	-0.0030 ± 1.294	232.48 ± 64.44
Conductivity	B_pair_ratio	5-NN	0.2971 ± 0.7939	218.32 ± 83.56
Conductivity	D_leave_HBA	Dummy mean	-367092.0 ± 1071646.5	348.78 ± 142.22
Conductivity	D_leave_HBA	1-NN	-138045.3 ± 316580.3	227.39 ± 240.11
Conductivity	D_leave_HBA	5-NN	-288393.1 ± 786848.9	249.84 ± 229.79
Conductivity	D_leave_HBD	Dummy mean	-4106.2 ± 10824.1	378.39 ± 183.34
Conductivity	D_leave_HBD	1-NN	-12557.8 ± 39691.0	329.29 ± 318.22
Conductivity	D_leave_HBD	5-NN	-13143.8 ± 41434.6	315.19 ± 287.86
Density	B_pair_ratio	Dummy mean	-0.0370 ± 0.0437	0.0868 ± 0.0080
Density	B_pair_ratio	1-NN	0.7620 ± 0.1125	0.0318 ± 0.0091
Density	B_pair_ratio	5-NN	0.7973 ± 0.0818	0.0323 ± 0.0078
Density	D_leave_HBA	Dummy mean	-15.39 ± 22.15	0.0765 ± 0.0256
Density	D_leave_HBA	1-NN	-36.68 ± 100.83	0.0729 ± 0.0514
Density	D_leave_HBA	5-NN	-35.21 ± 72.16	0.0708 ± 0.0350
Density	D_leave_HBD	Dummy mean	-10.75 ± 16.48	0.0853 ± 0.0610
Density	D_leave_HBD	1-NN	-17.73 ± 30.32	0.0914 ± 0.0403
Density	D_leave_HBD	5-NN	-19.57 ± 30.71	0.0993 ± 0.0457
Refractive index	B_pair_ratio	Dummy mean	-0.1123 ± 0.1240	0.0298 ± 0.0060
Refractive index	B_pair_ratio	1-NN	0.7271 ± 0.1268	0.0092 ± 0.0025
Refractive index	B_pair_ratio	5-NN	0.7724 ± 0.1053	0.0098 ± 0.0022
Refractive index	D_leave_HBA	Dummy mean	-38.62 ± 59.71	0.0409 ± 0.0170
Refractive index	D_leave_HBA	1-NN	-0.9119 ± 1.909	0.0136 ± 0.0069
Refractive index	D_leave_HBA	5-NN	-0.3587 ± 1.604	0.0130 ± 0.0088
Refractive index	D_leave_HBD	Dummy mean	-11.20 ± 14.80	0.0292 ± 0.0172
Refractive index	D_leave_HBD	1-NN	-8.457 ± 26.27	0.0122 ± 0.0056
Refractive index	D_leave_HBD	5-NN	-2.587 ± 7.362	0.0119 ± 0.0069
Surface tension	B_pair_ratio	Dummy mean	-0.1056 ± 0.1067	8.112 ± 1.705

Surface tension	B_pair_ratio	1-NN	0.6986 ± 0.1277	3.973 ± 1.400
Surface tension	B_pair_ratio	5-NN	0.7277 ± 0.1521	3.903 ± 1.295
Surface tension	D_leave_HBA	Dummy mean	-44.27 ± 130.01	8.023 ± 5.559
Surface tension	D_leave_HBA	1-NN	-13.04 ± 24.34	7.366 ± 3.382
Surface tension	D_leave_HBA	5-NN	-25.86 ± 53.40	7.470 ± 4.167
Surface tension	D_leave_HBD	Dummy mean	-11.79 ± 27.09	8.497 ± 5.157
Surface tension	D_leave_HBD	1-NN	-10.38 ± 20.82	8.069 ± 2.859
Surface tension	D_leave_HBD	5-NN	-9.312 ± 17.37	8.003 ± 3.036
Viscosity	B_pair_ratio	Dummy mean	-0.0189 ± 0.0205	2292.5 ± 635.52
Viscosity	B_pair_ratio	1-NN	0.1729 ± 0.2153	1258.9 ± 645.14
Viscosity	B_pair_ratio	5-NN	-1.413 ± 1.772	1654.6 ± 785.38
Viscosity	D_leave_HBA	Dummy mean	-1302.1 ± 3720.3	2863.7 ± 2673.7
Viscosity	D_leave_HBA	1-NN	-14627.6 ± 46169.4	4904.5 ± 6855.6
Viscosity	D_leave_HBA	5-NN	-6000.1 ± 18935.7	2961.1 ± 3277.3
Viscosity	D_leave_HBD	Dummy mean	-216.81 ± 441.54	1935.9 ± 1585.0
Viscosity	D_leave_HBD	1-NN	-4732.3 ± 14339.6	3208.2 ± 4992.9
Viscosity	D_leave_HBD	5-NN	-316.50 ± 719.31	2037.2 ± 3101.5

Only Protocol B and Protocol D, are retained for readability. Protocol C and full RMSE-level outputs are provided in the data repository. Values are reported as mean ± SD.

Table S8. Stage-specific model configurations and preprocessing used in the reproducible workflow.

Workflow stage	Model	Preprocessing	Key hyperparameters	Purpose
Diagnostic audit A–C	Ridge	Median imputation + standardization	alpha = 1.0	Linear reference model
Diagnostic audit A–C	ExtraTreesRegressor	Median imputation	n_estimators = 120; min_samples_leaf = 2; n_jobs = -1	Diagnostic leakage-aware non-linear model
Diagnostic audit A–C	HistGradientBoostingRegressor	Median imputation	max_iter = 150; learning_rate = 0.05; l2_regularization = 0.01	Diagnostic gradient-boosting benchmark
Protocol D extrapolative validation	Ridge	Median imputation + standardization	alpha = 1.0	Linear extrapolative reference
Protocol D extrapolative validation	ExtraTreesRegressor	Median imputation	n_estimators = 400; min_samples_leaf = 2; n_jobs = -1	Primary non-linear extrapolative benchmark
Protocol D extrapolative validation	HistGradientBoostingRegressor	Median imputation	max_iter = 300; learning_rate = 0.05; l2_regularization = 0.01	Gradient-boosting extrapolative benchmark
Feature ablation	Ridge	Median imputation + standardization	alpha = 1.0	Linear feature-group comparison
Feature ablation	ExtraTreesRegressor	Median imputation	n_estimators = 400; min_samples_leaf = 2; max_features = sqrt; n_jobs = -1	Primary feature-ablation model
Feature ablation	HistGradientBoostingRegressor	Median imputation	max_iter = 300; learning_rate = 0.05; l2_regularization = 0.01	Gradient-boosting feature-ablation model
Applicability-domain analysis	ExtraTreesRegressor	Median imputation; standardized distance space	n_estimators = 120; min_samples_leaf = 2; n_jobs = -1	Error-vs-distance analysis
SHAP interpretability	ExtraTreesRegressor	Median imputation	n_estimators = 300; min_samples_leaf = 2; n_jobs = -1	Global feature-importance analysis
Nearest-neighbor baseline	kNN regression	Median imputation + standardization	k = 1 and k = 5; uniform weights	Similarity-based baseline

Table S9. Literature comparison of representative DES ML/QSPR and mixture-QSPR studies. The table summarizes property scope, feature representation, model class, validation strategy, leakage/extrapolation relevance, and the relationship of each study to the present leakage-aware benchmark.

**Study**	**Focus / property**	**Data / system scope**	**Representation**	**Model class**	**Validation strategy**	**Leakage / extrapolation relevance**	**Relation to present work**
Halder et al. (2022)[1]	DES surface tension	Binary DES mixtures; surface-tension QSPR dataset	Mixture/property descriptors derived from HBA/HBD information	QSPR regression models	Mixture-level and compound-level validation discussed	Highlights need for chemically meaningful splits in DES mixture-property modeling	Property-specific QSPR study; present work extends the validation question across multiple DES properties and leakage-aware protocols
Odegova et al. (2024)[2]	DES melting temperature, density, and viscosity prediction platform	Open platform/database for DES property search and prediction	Supervised ML descriptors/features for DES property prediction	Supervised ML models	Platform-oriented predictive validation	Demonstrates utility of DES property prediction tools but does not center leakage-aware audit as the main objective	Complementary to platform development; present work focuses on audit-driven validation reliability and extrapolative behavior
Lemaoui et al. (2022)[3]	DES surface tension	Surface-tension records for DES systems	Molecular/physicochemical parameters	Artificial neural network	Training/test evaluation for predictive performance	Strong performance reported for a single target property; extrapolative leakage-aware validation is not the central focus	Representative prior DES ML property model; present work evaluates transferability under grouped and leave-component-out validation
Lemaoui et al. (2020)[4]	DES electrical conductivity	Conductivity QSPR dataset for DES systems	COSMO-RS sigma profiles as molecular descriptors	QSPR regression models	QSPR model validation	Uses richer quantum-chemical/COSMO-RS-derived descriptors for transport-property modeling	Supports discussion that transport properties may require interaction-aware or physics-informed descriptors beyond static RDKit features
Halder et al. (2025)[5]	DES heat capacity	Heat-capacity records for DES systems	COSMO-RS-based descriptors capturing detailed DES structural information	ML models including neural-network approaches	Predictive model validation and interpretability	Shows value of COSMO-RS descriptors and SHAP analysis for DES thermophysical properties	Supports future-representation discussion; present work benchmarks static descriptor limitations across multiple properties
Fu et al. (2026)[6]	Fluorinated gas solubility in DES	Gas-solubility prediction for DES systems	Hybrid thermodynamics-informed data-driven representation	Hybrid thermodynamics-informed ML/data-driven models	Application-focused model validation	Illustrates physics-informed strategies for DES-related prediction beyond purely static descriptors	Supports future-work direction toward hybrid/physics-informed representations for challenging DES properties

Oprisiu et al. (2013)[7]	Non-additive mixture properties	Mixture-property QSPR using OCHEM	Mixture descriptors and cheminformatics representations	QSPR / machine-learning models	Mixture and compound-level validation paradigms	Provides conceptual basis for distinguishing points-out, mixture-out, and compounds-out validation	Used to frame Protocols A relative to established mixture-QSPR validation concepts
Present work	Density, viscosity, conductivity, surface tension, refractive index	Frozen GOLD DES dataset spanning 2003-2026	Static descriptor-ratio-temperature representation	Ridge, ExtraTrees, HistGradientBoosting, same-dataset model-class benchmark	Leakage-aware Protocols A–D, including pair+ratio grouping, pair-out validation, and leave-component-out validation	Primary contribution is audit-driven evaluation of leakage sensitivity, applicability domain, and extrapolative transferability	Benchmark study quantifying where descriptor-based ML succeeds or fails under leakage-aware validation

Table S10. Same-dataset model-class benchmark under leakage-safe validation.

**Property**	**Target variant**	**Protocol**	**Model**	R^2 mean ± SD	MAE mean ± SD	RMSE mean ± SD	**MAPE (%) mean ± SD**	**MdAPE (%) mean ± SD**	**Folds**
Density	raw	B_pair_ratio	Ridge	0.819 ± 0.0268	0.0339 ± 0.00405	0.0492 ± 0.00664	3.04 ± 0.393	2.12 ± 0.560	5
Density	raw	B_pair_ratio	RandomForest	0.896 ± 0.0234	0.0228 ± 0.00374	0.0370 ± 0.00402	2.02 ± 0.343	1.14 ± 0.296	5
Density	raw	B_pair_ratio	ExtraTrees	0.905 ± 0.0278	0.0203 ± 0.00320	0.0352 ± 0.00422	1.82 ± 0.305	0.877 ± 0.209	5
Density	raw	B_pair_ratio	HistGradientBoosting	0.881 ± 0.0149	0.0238 ± 0.00169	0.0397 ± 0.00187	2.14 ± 0.195	1.03 ± 0.188	5
Density	raw	D_leave_HBA	Ridge	0.636 ± 0.241	0.0456 ± 0.0234	0.0641 ± 0.0384	4.20 ± 2.31	2.86 ± 0.929	5
Density	raw	D_leave_HBA	RandomForest	0.440 ± 0.496	0.0504 ± 0.0137	0.0664 ± 0.0106	4.58 ± 1.50	3.77 ± 1.36	5
Density	raw	D_leave_HBA	ExtraTrees	0.484 ± 0.454	0.0496 ± 0.0125	0.0647 ± 0.0115	4.54 ± 1.37	3.67 ± 1.30	5
Density	raw	D_leave_HBA	HistGradientBoosting	0.532 ± 0.238	0.0512 ± 0.00891	0.0666 ± 0.0107	4.63 ± 1.01	3.88 ± 0.774	5
Density	raw	D_leave_HBD	Ridge	0.498 ± 0.244	0.0523 ± 0.0130	0.0684 ± 0.0152	4.68 ± 1.08	3.73 ± 1.18	5
Density	raw	D_leave_HBD	RandomForest	0.630 ± 0.0813	0.0466 ± 0.0105	0.0612 ± 0.0161	4.16 ± 0.753	3.30 ± 0.770	5
Density	raw	D_leave_HBD	ExtraTrees	0.632 ± 0.120	0.0445 ± 0.0108	0.0604 ± 0.0170	4.04 ± 0.942	2.95 ± 0.593	5
Density	raw	D_leave_HBD	HistGradientBoosting	0.463 ± 0.221	0.0540 ± 0.0123	0.0718 ± 0.0177	4.88 ± 1.29	3.81 ± 1.40	5
Viscosity	raw	B_pair_ratio	Ridge	-0.443 ± 0.620	2961 ± 794	9726 ± 8523	5054 ± 2635	1165 ± 455	5
Viscosity	raw	B_pair_ratio	RandomForest	-0.0391 ± 0.529	1614 ± 774	8628 ± 8740	706 ± 724	59.1 ± 13.2	5
Viscosity	raw	B_pair_ratio	ExtraTrees	-0.245 ± 0.628	1774 ± 837	9068 ± 8549	918 ± 1306	57.1 ± 17.3	5
Viscosity	raw	B_pair_ratio	HistGradientBoosting	0.0649 ± 0.285	1932 ± 773	8467 ± 8777	1345 ± 639	245 ± 67.9	5
Viscosity	raw	D_leave_HBA	Ridge	-11.8 ± 20.6	4547 ± 1129	1.11E4 ± 8326	7715 ± 5078	3926 ± 3021	5
Viscosity	raw	D_leave_HBA	RandomForest	-1.09 ± 2.06	1712 ± 1411	7556 ± 9941	992 ± 525	132 ± 51.0	5
Viscosity	raw	D_leave_HBA	ExtraTrees	-1.73 ± 2.85	1931 ± 1200	8272 ± 9606	1142 ± 424	175 ± 45.1	5
Viscosity	raw	D_leave_HBA	HistGradientBoosting	-2.06 ± 3.15	2335 ± 1061	8202 ± 9501	2664 ± 1223	908 ± 598	5
Viscosity	raw	D_leave_HBD	Ridge	-4.77 ± 6.40	5010 ± 2008	1.36E4 ± 6741	7299 ± 3937	2456 ± 1524	5
Viscosity	raw	D_leave_HBD	RandomForest	-0.668 ± 1.05	2816 ± 1981	9732 ± 8571	2451 ± 2278	324 ± 293	5

Viscosity	raw	D_leave_HBD	ExtraTrees	-0.907 ± 1.97	2753 ± 1217	9498 ± 8287	2487 ± 1425	734 ± 729	5
Viscosity	raw	D_leave_HBD	HistGradientBoosting	-0.896 ± 1.54	3176 ± 1411	9766 ± 8203	3324 ± 1518	1055 ± 858	5
Conductivity	raw	B_pair_ratio	Ridge	-0.571 ± 1.23	421 ± 51.3	625 ± 173	2.12E5 ± 1.80E5	2215 ± 1666	5
Conductivity	raw	B_pair_ratio	RandomForest	0.116 ± 1.34	181 ± 112	384 ± 280	1.37E4 ± 1.97E4	80.8 ± 34.5	5
Conductivity	raw	B_pair_ratio	ExtraTrees	0.484 ± 0.559	177 ± 95.7	355 ± 227	2.22E4 ± 3.82E4	80.1 ± 50.3	5
Conductivity	raw	B_pair_ratio	HistGradientBoosting	0.248 ± 1.05	195 ± 113	377 ± 253	2.43E4 ± 3.29E4	170 ± 68.3	5
Conductivity	raw	D_leave_HBA	Ridge	-13.0 ± 11.5	612 ± 135	891 ± 251	4.19E5 ± 5.22E5	1.74E4 ± 2.79E4	5
Conductivity	raw	D_leave_HBA	RandomForest	-0.126 ± 0.820	217 ± 296	390 ± 458	3.67E4 ± 4.75E4	465 ± 803	5
Conductivity	raw	D_leave_HBA	ExtraTrees	-0.426 ± 1.13	236 ± 285	397 ± 442	4.01E4 ± 4.22E4	902 ± 1532	5
Conductivity	raw	D_leave_HBA	HistGradientBoosting	-0.415 ± 0.947	246 ± 292	421 ± 446	3.46E4 ± 3.82E4	881 ± 804	5
Conductivity	raw	D_leave_HBD	Ridge	-4.63 ± 9.01	639 ± 217	820 ± 275	4.38E5 ± 5.96E5	7433 ± 9994	5
Conductivity	raw	D_leave_HBD	RandomForest	-0.546 ± 1.52	275 ± 111	502 ± 207	5.36E4 ± 7.09E4	1592 ± 3114	5
Conductivity	raw	D_leave_HBD	ExtraTrees	-0.977 ± 2.27	281 ± 66.0	504 ± 109	3.71E4 ± 2.58E4	4134 ± 8394	5
Conductivity	raw	D_leave_HBD	HistGradientBoosting	-0.227 ± 0.963	266 ± 105	477 ± 217	8.19E4 ± 1.14E5	1687 ± 2685	5
Surface tension	raw	B_pair_ratio	Ridge	0.365 ± 0.694	4.48 ± 0.850	7.11 ± 3.48	12.0 ± 3.23	6.87 ± 2.51	5
Surface tension	raw	B_pair_ratio	RandomForest	0.782 ± 0.127	3.42 ± 1.43	4.60 ± 2.06	9.31 ± 4.47	6.41 ± 2.94	5
Surface tension	raw	B_pair_ratio	ExtraTrees	0.814 ± 0.0837	3.09 ± 0.947	4.26 ± 1.57	8.37 ± 2.96	5.15 ± 1.32	5
Surface tension	raw	B_pair_ratio	HistGradientBoosting	0.802 ± 0.0741	3.25 ± 1.12	4.41 ± 1.43	8.69 ± 3.18	6.24 ± 2.47	5
Surface tension	raw	D_leave_HBA	Ridge	-3.93 ± 7.90	9.13 ± 3.50	12.1 ± 3.78	24.2 ± 10.4	18.3 ± 11.5	5
Surface tension	raw	D_leave_HBA	RandomForest	0.144 ± 0.734	4.88 ± 1.74	6.66 ± 2.35	12.0 ± 5.46	8.35 ± 4.69	5
Surface tension	raw	D_leave_HBA	ExtraTrees	-0.0403 ± 1.07	5.39 ± 1.78	6.98 ± 2.33	13.7 ± 6.39	10.9 ± 4.85	5
Surface tension	raw	D_leave_HBA	HistGradientBoosting	0.0821 ± 0.745	5.37 ± 1.79	6.94 ± 2.26	13.2 ± 5.94	11.2 ± 4.78	5
Surface tension	raw	D_leave_HBD	Ridge	0.118 ± 0.427	5.86 ± 1.26	8.49 ± 2.48	15.2 ± 5.18	10.9 ± 5.56	5
Surface tension	raw	D_leave_HBD	RandomForest	0.163 ± 0.969	5.56 ± 1.92	6.95 ± 1.87	14.1 ± 4.50	12.0 ± 6.41	5
Surface tension	raw	D_leave_HBD	ExtraTrees	0.466 ± 0.401	4.96 ± 0.877	6.25 ± 0.818	13.0 ± 3.34	10.6 ± 3.19	5

Surface tension	raw	D_leave_HBD	HistGradientBoosting	0.109 ± 1.30	5.36 ± 2.43	6.63 ± 2.55	13.4 ± 5.78	11.5 ± 6.83	5
Refractive index	raw	B_pair_ratio	Ridge	0.714 ± 0.220	0.0115 ± 0.00498	0.0170 ± 0.00728	0.773 ± 0.335	0.477 ± 0.216	5
Refractive index	raw	B_pair_ratio	RandomForest	0.833 ± 0.0556	0.00921 ± 0.00191	0.0134 ± 0.00172	0.618 ± 0.130	0.463 ± 0.219	5
Refractive index	raw	B_pair_ratio	ExtraTrees	0.857 ± 0.114	0.00726 ± 0.00170	0.0115 ± 0.00328	0.487 ± 0.112	0.363 ± 0.117	5
Refractive index	raw	B_pair_ratio	HistGradientBoosting	0.841 ± 0.0612	0.00934 ± 0.00171	0.0130 ± 0.00159	0.625 ± 0.118	0.475 ± 0.173	5
Refractive index	raw	D_leave_HBA	Ridge	0.277 ± 0.886	0.0187 ± 0.00797	0.0256 ± 0.0131	1.26 ± 0.546	0.924 ± 0.352	5
Refractive index	raw	D_leave_HBA	RandomForest	0.747 ± 0.155	0.0136 ± 0.00310	0.0164 ± 0.00344	0.906 ± 0.204	0.855 ± 0.144	5
Refractive index	raw	D_leave_HBA	ExtraTrees	0.826 ± 0.0552	0.0113 ± 0.00253	0.0143 ± 0.00353	0.754 ± 0.168	0.704 ± 0.218	5
Refractive index	raw	D_leave_HBA	HistGradientBoosting	0.840 ± 0.0564	0.0111 ± 0.00404	0.0140 ± 0.00461	0.739 ± 0.269	0.697 ± 0.371	5
Refractive index	raw	D_leave_HBD	Ridge	0.423 ± 0.271	0.0146 ± 0.00491	0.0200 ± 0.00864	0.978 ± 0.339	0.693 ± 0.176	5
Refractive index	raw	D_leave_HBD	RandomForest	0.733 ± 0.0715	0.0104 ± 0.00358	0.0136 ± 0.00391	0.700 ± 0.247	0.603 ± 0.180	5
Refractive index	raw	D_leave_HBD	ExtraTrees	0.704 ± 0.127	0.0100 ± 0.00247	0.0138 ± 0.00248	0.672 ± 0.173	0.498 ± 0.140	5
Refractive index	raw	D_leave_HBD	HistGradientBoosting	0.669 ± 0.149	0.0114 ± 0.00583	0.0152 ± 0.00665	0.765 ± 0.403	0.548 ± 0.264	5

Representative linear and non-linear regression models were evaluated using the same descriptor–ratio–temperature feature space and grouped validation protocols. Values are rounded to three significant figures. For readability, the table reports Protocol B and leave-component-out Protocol D results; Protocol C and complete metric-level outputs are provided in the accompanying repository.

Table S11. Transport-property relative-error metrics for the primary ExtraTrees model.

Property	Evaluation setting	Protocol / split	Model	R^2 mean ± SD	MAE mean ± SD	RMSE mean ± SD	MAPE (%) mean ± SD	MdAPE (%) mean ± SD	Notes
Conductivity	Internal leakage-safe validation	B_pair_ratio	ExtraTrees	0.484 ± 0.559	177 ± 95.7	355 ± 227	2.22E4 ± 3.82E4	80.1 ± 50.3	MAPE may be inflated when target values are very small.
Conductivity	Internal leakage-safe validation	C_pair	ExtraTrees	0.337 ± 0.520	228 ± 91.4	431 ± 209	1.86E4 ± 1.43E4	244 ± 193	MAPE may be inflated when target values are very small.
Conductivity	Internal leakage-safe validation	D_leave_HBA	ExtraTrees	-0.426 ± 1.13	236 ± 285	397 ± 442	4.01E4 ± 4.22E4	902 ± 1532	MAPE may be inflated when target values are very small.
Conductivity	Internal leakage-safe validation	D_leave_HBD	ExtraTrees	-0.977 ± 2.27	281 ± 66.0	504 ± 109	3.71E4 ± 2.58E4	4134 ± 8394	MAPE may be inflated when target values are very small.
Conductivity	Recent-literature holdout	Train <2025; test 2025–2026	ExtraTrees	-687	238	389	4.14VE4	3560	Retrospective literature-holdout stress test; unseen pair fraction = 0.833.
Viscosity	Internal leakage-safe validation	B_pair_ratio	ExtraTrees	-0.245 ± 0.628	1774 ± 837	9068 ± 8549	918 ± 1306	57.1 ± 17.3	Relative errors reflect the broad dynamic range of viscosity.
Viscosity	Internal leakage-safe validation	C_pair	ExtraTrees	0.161 ± 0.216	1775 ± 1172	8048 ± 9308	778 ± 333	170 ± 135	Relative errors reflect the broad dynamic range of viscosity.
Viscosity	Internal leakage-safe validation	D_leave_HBA	ExtraTrees	-1.73 ± 2.85	1931 ± 1200	8272 ± 9606	1142 ± 424	175 ± 45.1	Relative errors reflect the broad dynamic range of viscosity.
Viscosity	Internal leakage-safe validation	D_leave_HBD	ExtraTrees	-0.907 ± 1.97	2753 ± 1217	9498 ± 8287	2487 ± 1425	734 ± 729	Relative errors reflect the broad dynamic range of viscosity.
Viscosity	Recent-literature holdout	Train <2025; test 2025–2026	ExtraTrees	-233	2104	4264	4043	1303	Retrospective literature-holdout stress test; unseen pair fraction = 0.900.

The table reports R^2, MAE, RMSE, MAPE, and MdAPE for conductivity and viscosity under internal leakage-safe validation and the recent-literature holdout stress test. Values are rounded to three significant figures. MAPE values should be interpreted cautiously for properties containing very small target values, especially conductivity; MdAPE is therefore reported alongside MAPE as a more robust relative-error summary.

Table S12. Recent-literature holdout performance of the primary ExtraTrees model.

Property	Train N	Holdout N	R^2	MAE	RMSE	MAPE (%)	MdAPE (%)	Holdout unique pairs	Unseen pair fraction
Density	852	172	0.669	0.0260	0.0358	2.46	1.60	11	0.91
Refractive index	439	72	0.823	0.0094	0.0106	0.61	0.62	4	1.00
Surface tension	355	114	-0.115	5.7520	6.4680	13.15	11.49	6	1.00
Conductivity	431	114	-687.376	237.5510	389.0100	41361.65	3559.77	6	0.83
Viscosity	797	147	-233.000	2103.5700	4263.7400	4042.81	1302.65	10	0.90

Models were trained on pre-2025 GOLD records and evaluated on 2025–2026 records withheld from training. Metrics include R^2, MAE, RMSE, mean absolute percentage error (MAPE), median absolute percentage error (MdAPE), and the fraction of HBA–HBD pairs in the holdout set not observed in the training pool. This analysis is a retrospective literature-holdout stress test and should not be interpreted as newly generated experimental validation.

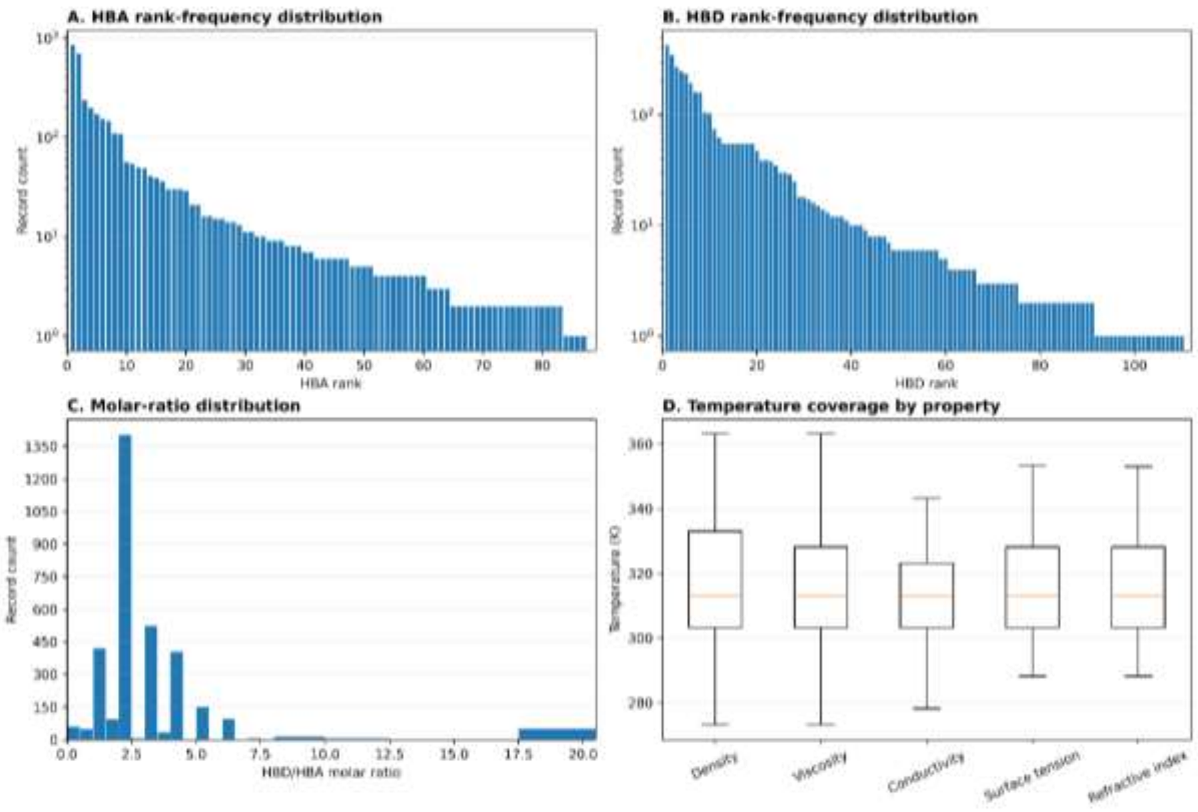

Figure S1. Dataset imbalance and coverage across DES systems. Rank-frequency distributions of hydrogen bond acceptor (HBA) and hydrogen bond donor (HBD) components, molar ratio distribution, and temperature coverage across properties. The distributions show a pronounced long-tail behavior and concentration around commonly studied compositions and near-ambient temperatures.

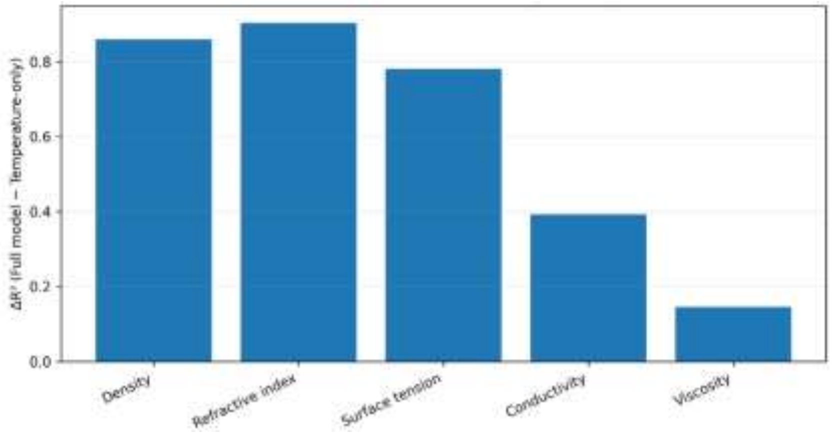

Figure S2. Performance gain relative to the temperature-only baseline. Difference in cross-validated R^2 between the full model and the temperature-only baseline under leakage-corrected validation.

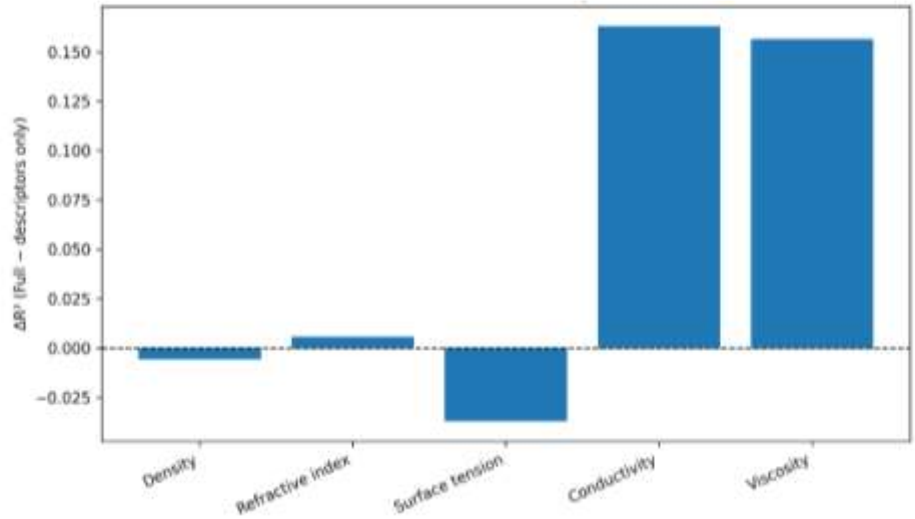

Figure S3. Incremental contribution of the full feature representation. Change in predictive performance (ΔR^2) between descriptor-only and full feature models, highlighting the relative contribution of composition and temperature features across properties.

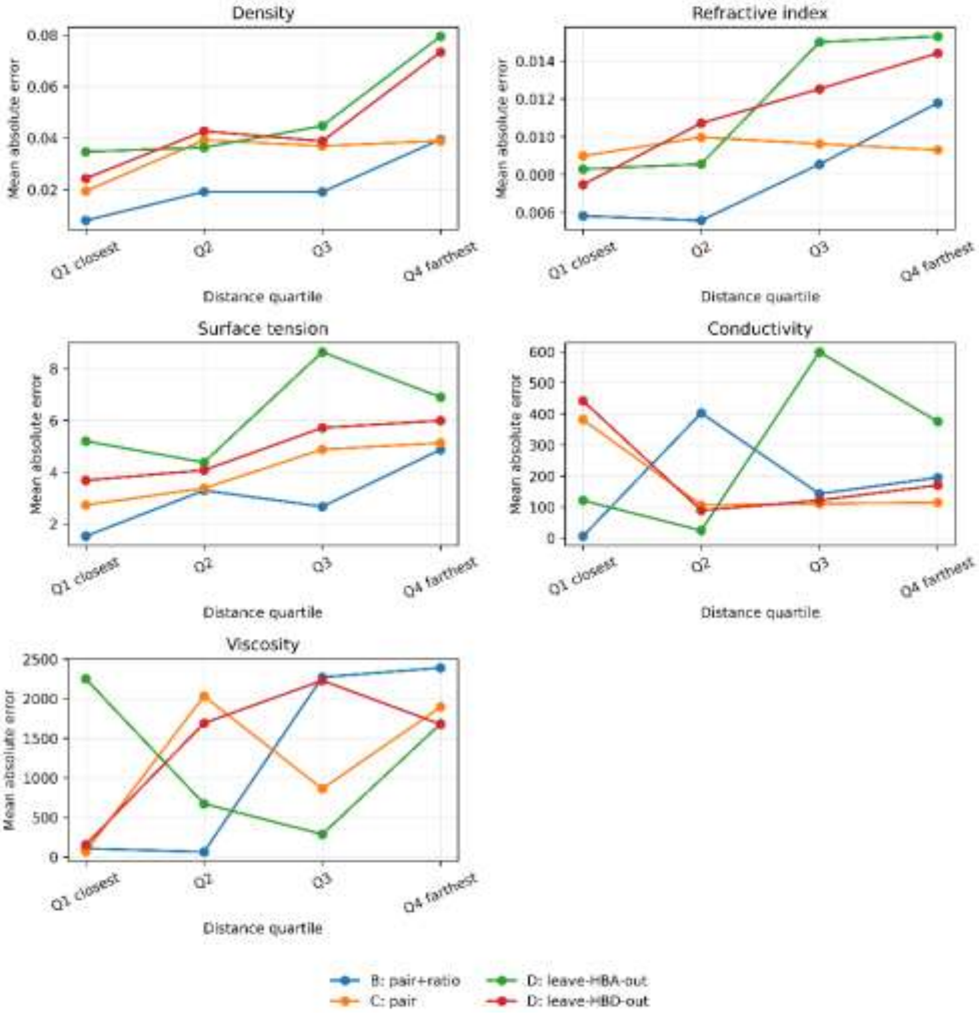

Figure S4. Applicability-domain analysis based on feature-space distance. Prediction error is evaluated as a function of distance to the training data and summarized by distance quartiles. Q1 represents samples closest to the training domain, whereas Q4 represents the farthest samples. Error generally increases with distance from the training domain, with the strongest degradation observed for electrical conductivity and viscosity.

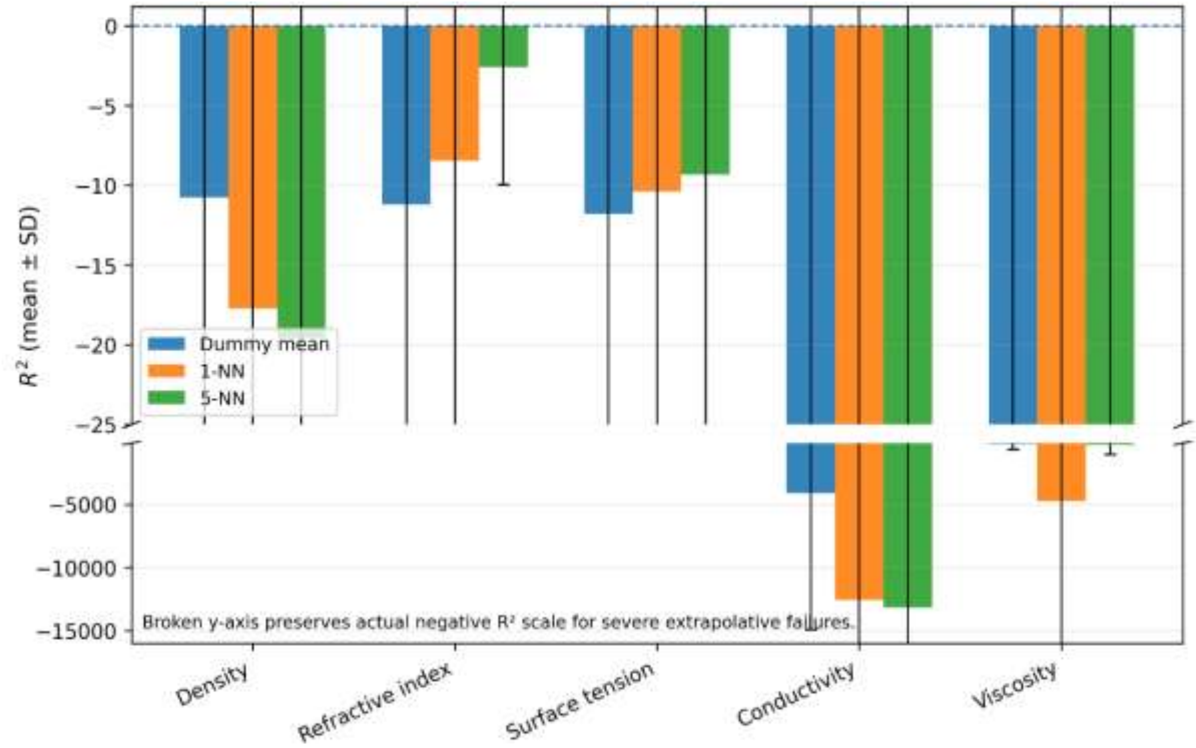

Figure S5. Nearest-neighbor baseline performance under extrapolative validation. Comparison of dummy, 1-NN, and k-NN models under leave-component-out conditions. Strongly negative R^2 values for conductivity and viscosity indicate that local similarity in feature space is insufficient for reliable prediction outside the training domain. The broken y-axis is used to preserve visibility of both moderate and strongly negative R^2 values.

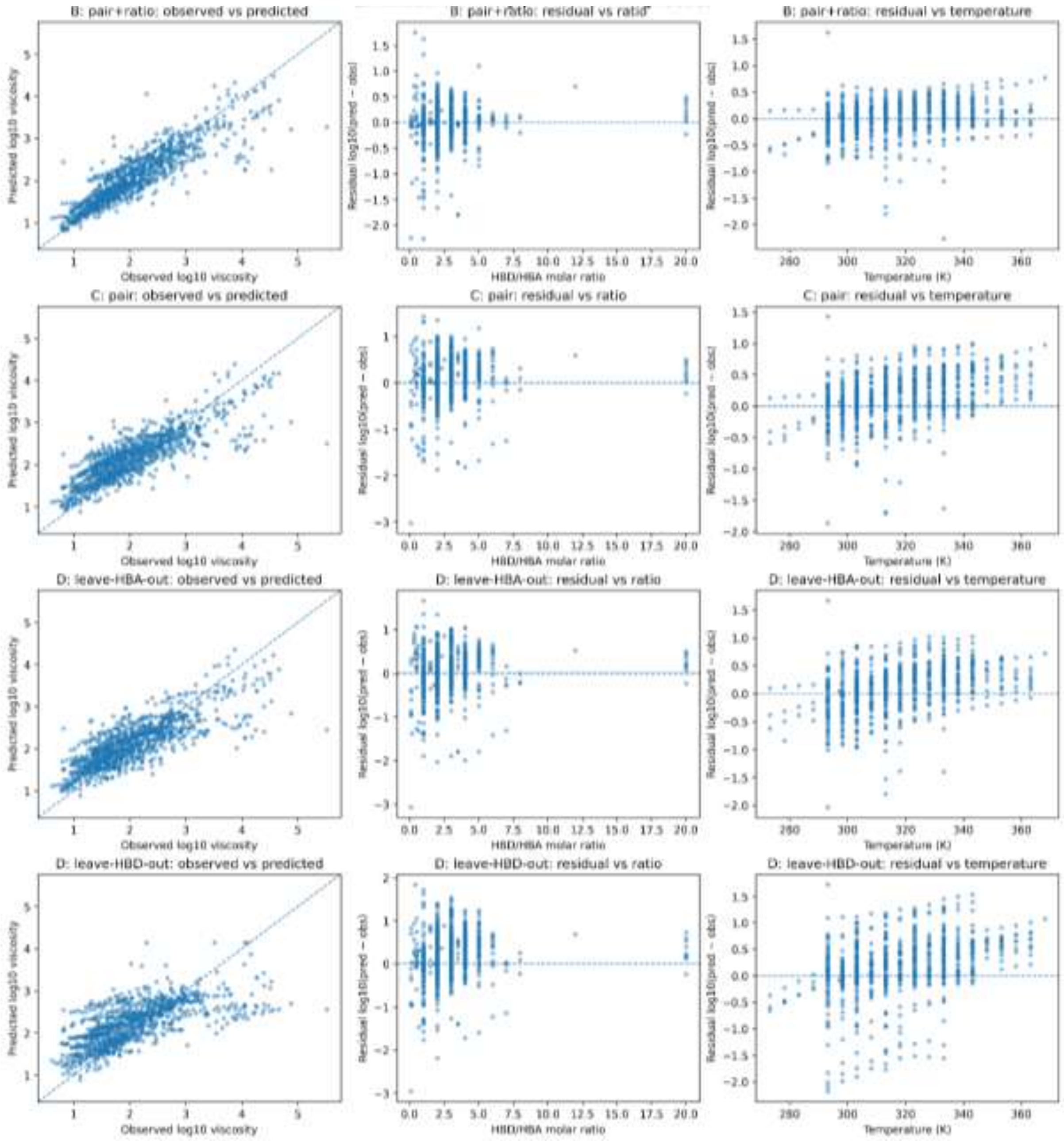

Figure S6. Extended viscosity prediction diagnostics. Observed-versus-predicted viscosity plots and residual distributions across validation protocols. Residuals are examined as a function of molar ratio and temperature, revealing systematic deviations and strong degradation under extrapolative leave-component-out validation.

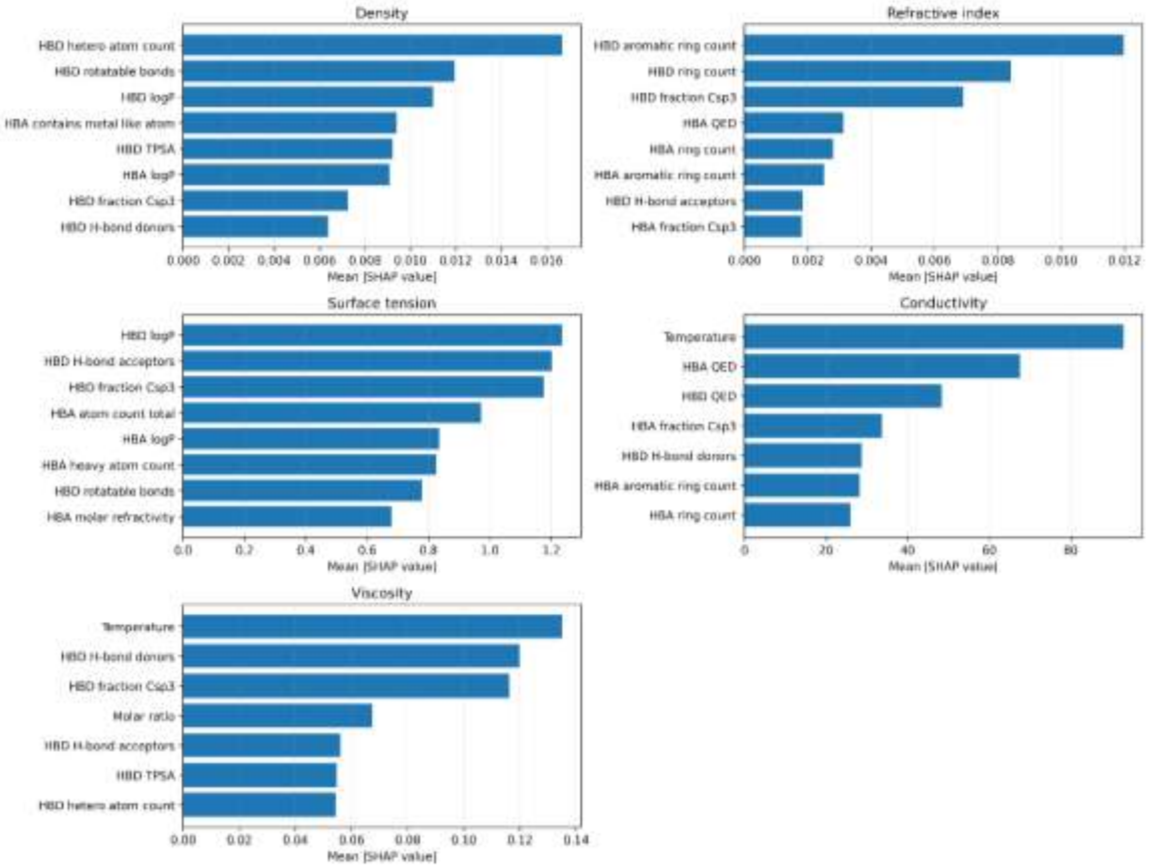

Figure S7. SHAP-based feature importance summary under leakage-corrected validation. Mean absolute SHAP values are shown for each property using the descriptors–ratio–temperature feature set. Temperature consistently emerges as the dominant predictor, followed by selected HBD descriptors, with strong variation across properties.
